# The Impact of COVID-19-Related Mitigation Measures on the Health and Fitness Status of Primary School Children in Austria: A Longitudinal Study with Data from 708 Children Measured before and during the Ongoing COVID-19 Pandemic

**DOI:** 10.3390/sports10030043

**Published:** 2022-03-11

**Authors:** Gerald Jarnig, Reinhold Kerbl, Mireille N. M. van Poppel

**Affiliations:** 1 Institute of Human Movement Science, Sport and Health, University of Graz, 8010 Graz, Austria; mireille.van-poppel@uni-graz.at; 2 Department of Pediatrics and Adolescent Medicine, LKH Hochsteiermark, 8700 Leoben, Austria; reinhold.kerbl@kages.at

**Keywords:** COVID-19, children, school, body mass index, waist-to-height ratio, weight classification, physical fitness, health-related fitness, cardiorespiratory endurance

## Abstract

The COVID-19-related closing of schools and sport facilities resulted in major changes to daily routines worldwide. It was the aim of this study to investigate the impact of COVID-19-related mitigation measures on the health and fitness status of primary school children in Austria. Seven hundred and eight primary school children (7–10 years old) participated in the longitudinal study. Data on height, weight, waist circumference, and fitness were collected before (September 2019) and during the course of the COVID-19 pandemic (June 20, September 20, March 21, June 21). A significant increase in EQUI BMI_AUT_ (η_p_^2^ = 0.087) and significant changes (η_p_^2^ = 0.355) in waist circumference were found. Cardiorespiratory endurance (η_p_^2^ = 0.440) and action speed (η_p_^2^ = 0.221) decreased dramatically following lockdowns/school closures. In contrast, muscle strength showed no significant changes. The COVID-19-related mitigation measures intended to contain a communicable disease resulted in an acceleration of the pre-existing pandemic of overweight and obesity. The adverse combination of increasing BMI and the loss of physical fitness is likely to result in long-term negative effects on the health status of growing and developing individuals. Health professionals should therefore not only support further longitudinal observations of this “non-communicable disease” but also support intervention programs to reverse this worrying side-effect of COVID-19-associated containment policies.

## 1. Introduction

The lifelong benefits and positive health outcomes of adequate physical activity and related improvements in physical fitness during childhood and adolescence have been reported in numerous studies [1,2,3,4,5,6,7,8]. Similarly, economic and social benefits have been reported to be directly related to physical activity, improved fitness, and psychological well-being [9,10,11,12,13]. An American study showed that low cardiorespiratory fitness in youth leads to a three-to-six-fold increased risk of developing metabolic syndrome, as well as diabetes and hypertension, as compared to that observed in youth with a high level of cardiorespiratory fitness [14].

However, regardless of these multiple benefits, a secular trend of increasing body mass index (BMI) [7,15] has been observed in children and adolescents over many years. In the 1980s and 1990s, the increase in BMI was accompanied by a decline in physical fitness [16,17,18,19,20,21,22]. However, due to increasing awareness of the far-reaching problem of decreased cardiorespiratory endurance in children and the specific interventions taken to counteract this, cardiorespiratory endurance values in children and adolescents have remained relatively stable since the beginning of this millennium [17,19,23].

Studies have reported that a poor cardiorespiratory endurance is positively correlated with increased BMI and abdominal circumference and favors the development of metabolic syndrome over the lifespan [24,25,26]. Subsequently, cardiorespiratory endurance has also been characterized as a strong health marker, as increased cardiorespiratory endurance is associated with healthier bodyweight and waist circumference [27]. Several different national (e.g., in Germany [28], Slovenia [29], the USA [30], Australia and Finland [31], and South Africa [32]) and international (e.g., the “Daily Mile” project (www.thedailymile.co.uk, 5 March 2022) [33,34,35,36]) interventions have been implemented to increase public awareness, with outcomes showing a trend reversal in physical fitness levels [28,29].

With the beginning of the worldwide COVID-19 pandemic and the associated restrictions on physical activity and temporary bans (mostly during lockdown phases) on organized sports in general and in schools, the already serious obesity pandemic underwent a worrisome acceleration [37,38,39,40,41] and health-related fitness scores showed dramatic decreases [42,43,44,45,46,47].

In the studies cited, the effects of the COVID-19 pandemic on bodyweight and fitness were observed over a short period of time.

The aim of the present study was to report changes in the anthropometric characteristics and health-related fitness of primary school children during the ongoing COVID-19 pandemic over an extended period and to investigate whether there were different trends in subgroups of sex (girls/boys) or region (urban/rural).

## 2. Materials and Methods

### 2.1. Design

Originally, this study was designed as a randomized controlled trial to investigate the effects of a physical education (PE) teaching intervention on the health and fitness of primary school children in the districts of Klagenfurt (urban and rural), Austria using a newly created Austrian fitness monitoring system for school children (AUT FIT) [48]. The intervention had to be interrupted due to COVID-19 restrictions. However, the follow-up fitness measurements planned for June 2020, September 2020, and June 2021 were carried out under strict hygiene regulations. Furthermore, additional anthropometric data could be collected in March 2021. All data were collected by a team of sports scientists during regular school hours between 8:00 and 11:30 a.m. A total of two school hours were needed: in the first lesson, anthropometrics, cardiorespiratory fitness and strength were measured; the remaining tests took place during the second hour. Before each test, the children had enough time to rest (more than 5 min) in order to complete the test in a well-rested state. Data were analyzed in a longitudinal study, and we assessed the impact of COVID-19 mitigation measures on the fitness and health status of primary school children. The study was registered in the German Clinical Trials Registry (ID DRKS00023824) and approved by the Research Ethics Committee of the University of Graz, Styria, Austria (GZ. 39/23/63 ex 2018/19), on 28 January 2019.

### 2.2. Selection of Schools and Participants

**Figure 1:**
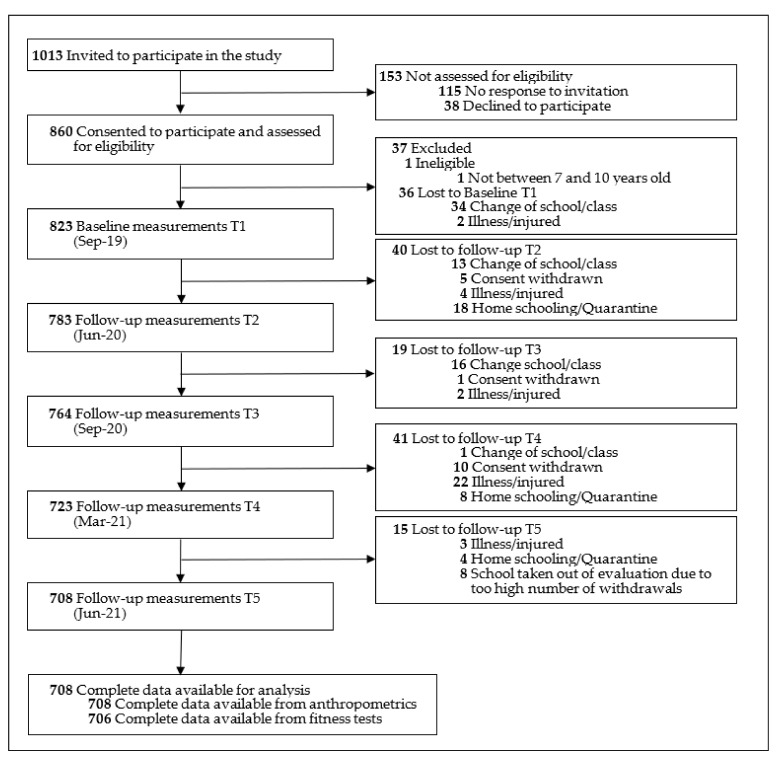
Flow diagram.

Using a number randomization generator, 12 out of 39 primary schools in the district of Klagenfurt (Austria) were selected. The inclusion criteria were that children participating in the study had no physical limitations and were between 7 and 10 years old at baseline. In spring 2019, all 1013 children attending the first or second grade at that time in one of the 12 schools were invited to participate in the study. A total of 860 (84.9%) legal guardians gave their written consent for the participation of their children (Figure 1).

### 2.3. Procedures

A testing team of sports scientists and PE teachers was recruited and trained to carry out the data collection. Baseline measurements (T1) were carried out in September 2019, and the PE intervention started in October 2019. In the intervention group, school-based PE lessons were led by external coaches and carried out according to the school curriculum, whereas in the control group PE lessons were led by their regular class teacher, with the number of lessons the same. In March 2020, the PE intervention had to be interrupted due to the COVID-19 pandemic spreading worldwide. After the first lockdown phase (March 2020 until the end of May 2020), the planned follow-up measurements could be carried out in June 2020 (T2) under strict hygiene regulations. Anthropometric data could be assessed according to the planned testing protocol. Fitness tests included in AUT FIT were also carried out; however, due to the changed testing conditions (testing outdoors, with shoes and everyday clothes) the fitness data were excluded from the longitudinal assessment. Further scheduled follow-up measurements in September 2020 (T3) and June 2021 (T5) could be performed according to the original test protocol (except for the V sit-and-reach test) in compliance with the existing COVID-19 and hygiene regulations. Additionally, anthropometric data were assessed after the second lockdown phase (end of October 2020 until end of March 2021) in March 2021 (T4).

To visualize the restrictions imposed by the government over the period of interest, the Oxford COVID-19 Government Response Tracker (OxCGRT) provides the internationally comparable stringency level for Austria (Tables S1 and S2, Figure S1B). Based on the OxCGRT classification, a more detailed, self-developed stringency level for the impact of a pandemic response on children over time can be viewed (Tables S1 and S2, Figure S1A). The detailed description of this classification method based on the Austrian legal regulations, which can be viewed in the Federal Law Gazette [49], can be found in the Supplementary Materials (Tables S1 and S2, Figure S1).

### 2.4. Outcomes

For this study, the primary outcome was the impact of lockdown phases on the fitness and health status of primary school children.

Secondary analyses were conducted for subgroups by gender, school location (urban vs. rural), and school grade (second or third grade in the 2019/20 school year). The Eurostat definition of urban–rural typology [50] was used, based on the number of inhabitants per km^2^. Urban areas were defined as areas with more than 300 inhabitants per km^2^, whereas the other areas were classified as rural. The demographic data of the municipality where the school was located were used to classify urban and rural schools [51,52].

#### 2.4.1. Anthropometrics Data

Bodyweight (kg) was measured to the nearest 0.1 kg using a Bosch PPW4202/01 (Bosch home appliances, Vienna, Austria) body scale. Height (cm) was measured to the nearest 0.1 cm using a SECA 213 stadiometer, and the waist circumference (cm) of the children was measured to the nearest 0.1 cm using a GIMA 27343 (GIMA Professional Medical Products, Milano, Italy) body tape measure.

The crude BMI (body weight in kg, divided by height squared in meters) and the waist-to-height ratio (WHtR, dividing waist circumference in cm by height in cm) were calculated.

The assessment of anthropometric data was performed according to the methods described in AUT FIT [48], resulting in national [53] weight classification categories and health risk estimation categories (for more details, see AUT FIT monitoring tool one and two) [48].

#### 2.4.2. Physical Fitness

In the pilot study for the development of AUT FIT [48], some modifications in the fitness-related monitoring tools of health-related and motor fitness were recommended. Thus, the data for the ruler drop test and jumping sideways were excluded in this study. Due to COVID-19-related increased hygiene regulations, the original test protocol could not be completed within the timeframe, and the V sit-and-reach test was carried out parallel to classroom teaching in T3 and T5 without any warm-up period. As this modified test protocol likely influenced the results, these data were excluded from the longitudinal assessment.

In the current study, the fitness tests were assessed individually using international reference values, but for the balance test described in AUT FIT [48] no reference or standardized values are available; therefore, the results of the balance tests were excluded from the longitudinal assessment.

Thus, the 6 min run (6MR; for cardiorespiratory endurance), standing long jump (SLJ; for muscular strength), medicine ball throw (MB1 kg; for muscular strength), and 4-m × 10-m shuttle run (4 × 10 SHR; for action speed) were used.

The 6MR: The children were instructed to run continuously around a rectangle (6-m × 18-m) for 6 min and to complete a maximum possible distance in this time.

The SLJ: From a starting line, the children had to jump as far as possible using both legs. Measurement of the shortest distance between the starting line and the contact of the child’s heel with the ground was carried out with a tape measure to the nearest cm. The furthest of three scoring attempts was included in the overall assessment.

The MB1 kg: From a starting line, the children had to push a 1 kg medicine ball, which was held with both hands and touched the chest, as far forward as possible. Measuring the shortest distance between the starting line and the ball’s contact with the ground was carried out with a tape measure to the nearest centimeter. The furthest of two scored attempts was included in the overall assessment.

The 4 × 10 SHR: To perform the 4 × 10 SHR, two lines (start line and turning line) were marked on the floor at a distance of 10 m from each other. Two objects (O1 and O3) were placed behind the turning line and an easily pickable object (O2) was placed in front of the start line. The children had to run from the start line across the turning line, pick up O1, run back over the start line, and set O1 down. Then they picked up O2, ran across the turning line, put O2 down, picked up O3, and ran with it over the starting line. The children were instructed to complete this test as quickly as possible. Two scoring attempts were made, and the time was measured with a stopwatch to the nearest 0.01 s. Each child had two attempts, and the fastest run was scored.

The validity and reliability of these four fitness tests have been described in detail before and have been reported to be suitable for use in field tests [48].

### 2.5. Standardization and Classification

Continuous variables are reported as means (M) and standard deviations (SD) and categorical variables as absolute values (n) and percentages (%) for descriptive statistics. No imputation of the data was performed.

#### 2.5.1. Anthropometric Data

Standardization and classification of weight and the estimation of health risk was carried out according to the methodology described in the pilot study for AUT FIT [48].

In short, national reference values were used for BMI standardization and weight classification [53]. Absolute BMI values were converted to EQUI BMI values (in this paper referred to as EQUI BMI_AUT_) using the procedure described by Mayer et al. 2015 [53] (based on Cole et al. 2000 [54]). EQUI BMI curves can be used to project actual BMI to cutoff values at the age of 18 years, which is relevant to classifying children’s weight into five categories (underweight EQUI BMI_AUT_ < 18.5 kg/m^2^, normal weight EQUI BMI_AUT_ 18.5 to 25.0 kg/m^2^, overweight EQUI BMI_AUT_ ≥ 25.0 kg/m^2^, obese EQUI BMI_AUT_ ≥ 30.0 kg/m^2^, and morbidly obese EQUI BMI_AUT_ ≥ 35.0 kg/m^2^).

The cutoff values of 0.5 and 0.6 were used to classify the WHtR and to enable a more comprehensive estimation of health risk, and the WHtR was divided into no (<0.5), increased (0.5 to 0.6), and high (≥0.6) health risks.

#### 2.5.2. Physical Fitness

To compare the results (raw scores) of the fitness tests with established reference values, standard deviation scores (SDS) and traditional z-scores (z-values) were created based on age- and sex-specific reference values. Since no national reference values are available for this age group, international reference values were used. For the 6MR and the SLJ, the most recent German percentile tables of the Düsseldorf model ([28]; collected 2011–2018) were used, and for the 4 × 10 SHR, Portuguese norm values [55] from the motor competence assessment (MCA) from 2019 were used. For these international references, calculations were performed based on the LMS method [56] using the German (DüMo) and Portuguese (MCA) reference tables. For the MB1 kg, German normative values [57] of the Karlsruhe Test System for Children (KATS-K) from 2001 were used and z-values were calculated via a traditional z-value standardization method.

Based on the methodology described in AUT FIT [48], the SDS or z-scores of each fitness test were transformed into a nine-point score (STA9). For the classification of each test the poorest performance was represented by one point and the best performance by nine points.

#### 2.5.3. Changes over Time

The changes in EQUI BMI_AUT_, WHtR, 6MR SDS (DüMo), SLJ SDS (DüMo), MB1 kg z-value (KATS-K), and 4 × 10 SHR SDS (MCA) were analyzed over the observation period by means of mixed-design analyses of variance (ANOVAs) for sex, school location, and school grade, and the time points September 2019 (T1), June 2020 (T2), September 2020 (T3), March 2021 (T4), and June 2021 (T5) for anthropometrics, and September 2019, September 2020, and June 2021 for fitness. Harley’s F-max test was used to test for homogeneity. The Greenhouse–Geisser adjustment was used to correct sphericity violations. For ANOVAs, partial eta squared (η_p_^2^) was used to determine the size of the effect (≥0.01 = small, ≥0.06 = medium, ≥0.14 = large) [58], and only small effects (at least) were considered relevant.

Changes over time in the distribution of the BMI, WHtR, and fitness classification categories were tested with the Friedman test. Post hoc tests were performed with the Wilcoxon signed-rank test.

All tests were two-tailed, with a *p*-value < 0.05 considered statistically significant. Bonferroni correction was used for post hoc tests.

All statistical calculations were performed using SPSS Version 27 (IBM Corp. Released 2020. IBM SPSS Statistics for Windows, Armonk, NY, USA: IBM Corp.).

## 3. Results

In September 2019, 823 children participated in the baseline measurements. One hundred and fifteen children did not participate in all five measurement time points of anthropometric data collection and were excluded from the analyses. One hundred and seventeen children did not participate in all three measurement time points, in which fitness data for the 6MR, SLJ, and 4 × 10 SHR were collected. The German reference values available for the assessment of the MB1 kg were only established for ages 6 to 10 years; therefore, the performance of 44 children aged > 10 years at test phase 5 could not be assessed. In total, complete anthropometric data were available for 708 children and fitness-related data for 706 (6MR, SLJ, 4 × 10 SHR) and 662 (MB1 kg) children, respectively (Figure 1). The included study population and the group lost to follow-up were compared on the variables of age, sex, school location, BMI, and abdominal circumference. Children who could not be followed up were more often female and had poorer results in the 6 min run and 4-m × 10-m shuttle run, but no differences in anthropometric data or muscle strength were observed (Table S3).

In the sample included in the analyses, the mean age at baseline was 8.3 ± 0.7 years (range: 7 to 10 years), 350 (49.4%) were girls, 424 (59.9%) children attended schools in urban areas, and at baseline 345 (48.7%) children were attending the second grade of primary school (Table 1 and Tables S4 and S5).

### 3.1. Change in BMI

**Figure 2:**
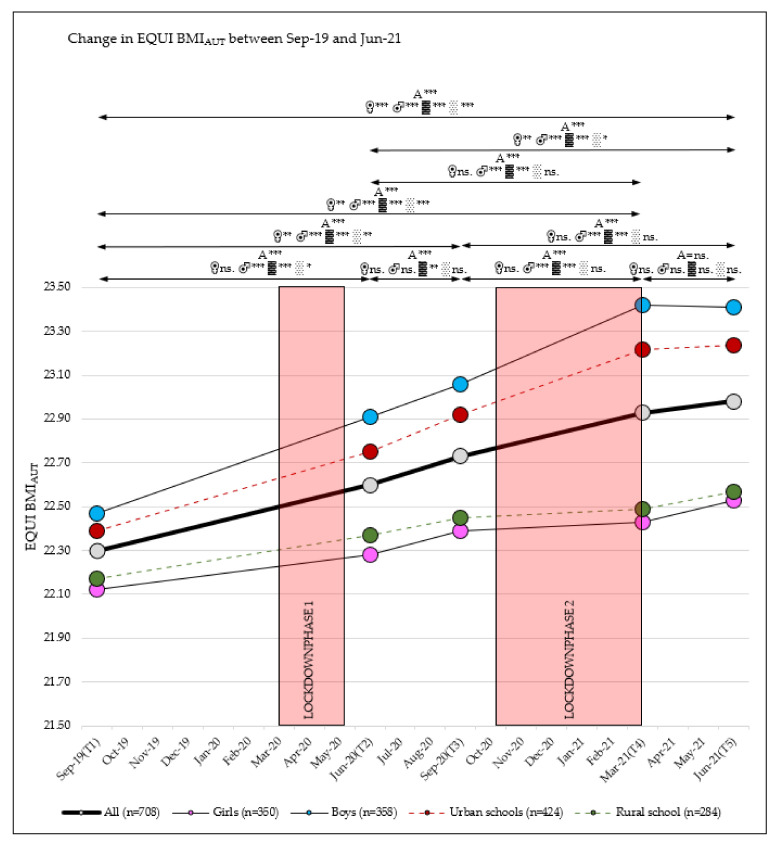
EQUI BMI_AUT_ ratios over five test phases (T1, T2, T3, T4, T5); A = all, ♀ = girls, ♂ = boys, ▓ = urban, ░ = rural; significance level *p*-value: * = *p*-value ≤ 0.05, ** = *p*-value ≤ 0.01, *** = *p*-value ≤ 0.001, ns. = *p*-value not significant.

Between September 2019 and June 2021, the EQUI BMI_AUT_ level continuously increased from 22.30 to 22.98 (main effect time: η_p_^2^ = 0.087; *p* < 0.001). Boys showed a significantly larger increase in BMI than girls (+0.94 (95% CI, 0.77–1.12) versus +0.41 (95% CI, 0.29–0.53)) and children attending urban schools showed a larger increase in BMI than children attending rural schools (+0.86 (95% CI, 0.71–1.01) versus +0.40 (95% CI, 0.26–0.55)) with small interaction effects over time (time × sex: η_p_^2^ = 0.022; *p* < 0.001; time × school location: η_p_^2^ = 0.013; *p* < 0.001) (Table 2 and Table 3 and Table S6, Figure 2).

No differences were found between children attending grades 2 and 3 in the 2019/20 school year (Table 3).

### 3.2. Change in WHtR

**Figure 3:**
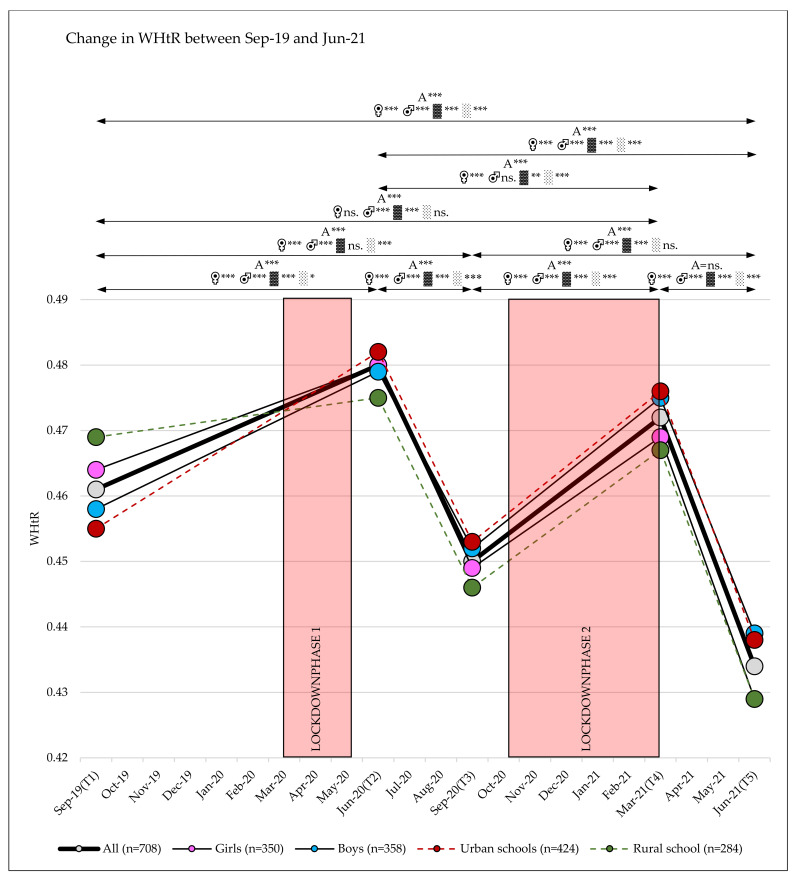
Waist-to-height ratios over five test phases (T1, T2, T3, T4, T5); A = all, ♀ = girls, ♂ = boys, ▓ = urban, ░ = rural; Significance level *p*-value: * = *p*-value ≤ 0.05, ** = *p*-value ≤ 0.01, *** = *p*-value ≤ 0.001, ns. = *p*-value not significant.

The WHtR increased significantly in each of the lockdown phases (between T1 and T2, respectively, and T3 and T4), and subsequently decreased significantly in the restriction relaxing phases (between T2 and T3, respectively, and T4 and T5) (Table 2, Figure 3). Between September 2019 and June 2021, the WHtR levels changed significantly, with large effects between the single test phases (main effect time: η_p_^2^ = 0.355; *p* < 0.001) and small interaction effects over time (time × sex: η_p_^2^ = 0.014; *p* < 0.001; time × school location: η_p_^2^ = 0.037; *p* < 0.001) (Table 2 and Table 3 and Table S7, Figure 3).

No differences were found between children attending grade 2 or 3 in the 2019/20 school year (Table 3).

### 3.3. Change in Fitness

**Figure 4:**
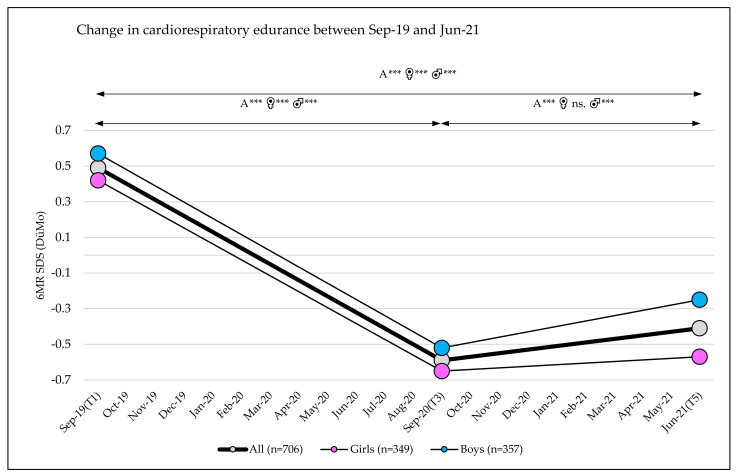
Six-minute run standard deviation scores over three test phases (T1, T3, T5); A = all, ♀ = girls, ♂ = boys, 6MR = 6 min run, SDS = standard deviation score; significance level *p*-value: *** = *p*-value ≤ 0.001, ns. = *p*-value not significant.

Cardiorespiratory fitness (6MR SDS (DüMo)) showed significant changes, with a large effect over the observation period (T1 to T5: main effect time: η_p_^2^ = 0.440; *p* < 0.001). An extreme decrease was observed between September 2019 and September 2020 (T1 = 0.49 to T3 = −0.59 (−1.08 (95% CI, −1.01–−1.15)); *p* < 0.001), followed by a slight increase between September 2020 and June 2021 (T3 = −0.59 to T5= −0.41 (+0.18 (95% CI, 0.11–0.24)); *p* < 0.001) (Table 3 and Table 4 and Table S8, Figure 4). No differences were found between subgroups for sex, school location, or school grade (Table 3).

Muscle strength (SLJ SDS (DüMo) and MB1 kg z-values (KATS-K)) showed significant changes, with small effects over the observation period (T1 to T5: main effect time: SLJ: η_p_^2^ = 0.031; *p* < 0.001; MB1 kg: η_p_^2^ = 0.011; *p* < 0.001) and with small interaction effects over time (SLJ SDS DüMo: time × school location: η_p_^2^ = 0.017; *p* < 0.001; time × school grade: η_p_^2^ = 0.048; *p* < 0.001; MB1 kg z-values (KATS-K): time × sex: η_p_^2^ = 0.013; *p* < 0.001) (Table 3 and Table 4 and Table S8).

**Figure 5:**
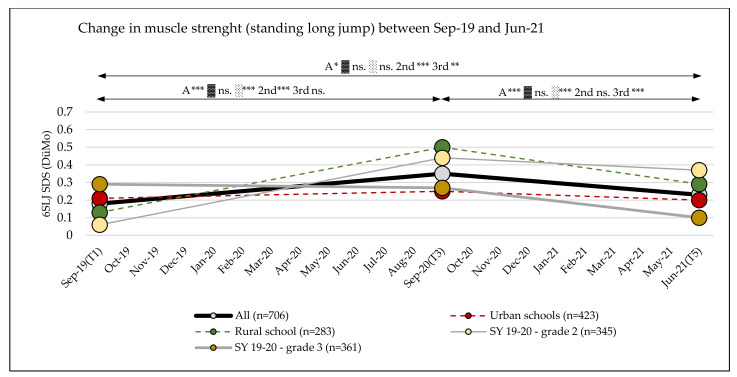
Standing long jump standard deviation scores over three test phases (T1, T3, T5); A = all, ▓ = urban, ░ = rural, 2nd = second grade in the 2019/20 school year, 3rd = third grade in the 2019/20 school year; SLJ = standing long jump, SDS = standard deviation score; significance level *p*-value: * = *p*-value ≤ 0.05, ** = *p*-value ≤ 0.01, *** = *p*-value ≤ 0.001, ns. = *p*-value not significant.

In SLJ, an increase was seen between September 2019 and September 2020 (SLJ SDS (DüMo) = +0.18 (95% CI, 0.11–0.24)). This increase was more pronounced in children attending rural schools (0.37 (95% CI, 0.26–0.47)) and in the second grade in the 2019/20 school year) (0.38 (95% CI, 0.29–0.47)) than in children attending urban schools (0.05 (95% CI, −0.03–0.13)) or the third grade in school year 2019/20 (−0.02 (95% CI, −0.10–0.07]) (Table 3 and Table 4, and Table S8, Figure 5). In the following period between September 2020 and June 2021, a decrease in performance was observed in all assessed subgroups, which was more pronounced in children attending rural schools (−0.21 (95% CI, –0.30–−0.12)) and the third grade in the 2019/20 school year (−0.17 (95% CI, −0.24–−0.09)) than in children attending urban schools (−0.06 (95% CI, −0.13–0.01]) and the second grade in the 2019/20 school year (−0.07 (95% CI, −0.16–0.02)) (Table 3 and Table 4, and Table S8, Figure 5). No differences were found between subgroups for sex (Table 3).

**Figure 6:**
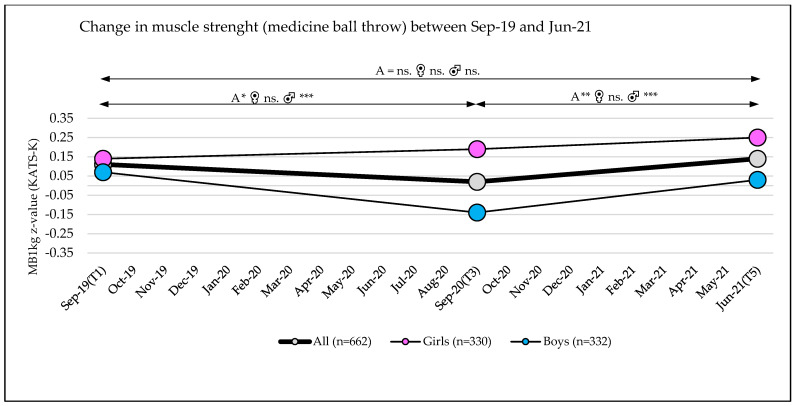
Medicine ball throw (1 kg) z-values over three test phases (T1, T3, T5); A = all, ♀ = girls, ♂ = boys, MB1 kg= medicine ball throw (1 kg), significance level *p*-value: * = *p*-value ≤ 0.05, ** = *p*-value ≤ 0.01, *** = *p*-value ≤ 0.001, ns. = *p*-value not significant.

Analysis of MB1 kg z-values (KATS-K) showed a significant decrease between September 2019 and September 2020 (−0.08 (95% CI, −0.15–−0.02)). This decrease was more pronounced in boys (−0.21 (95% CI, −0.30–−0.13)) than in girls (0.05 (95% CI, −0.05–−0.14)). In the subsequent period between September 2020 and June 2021, an increase in performance was observed (0.11 (95% CI, 0.05–0.18)) that was again more pronounced in boys (0.17 (95% CI, 0.080.25)) than in girls (0.06 (95% CI, −0.03–0.15)) (Table 3 and Table 4, and Table S8, Figure 6). No differences were found between subgroups for school location or school grade (Table 3).

**Figure 7:**
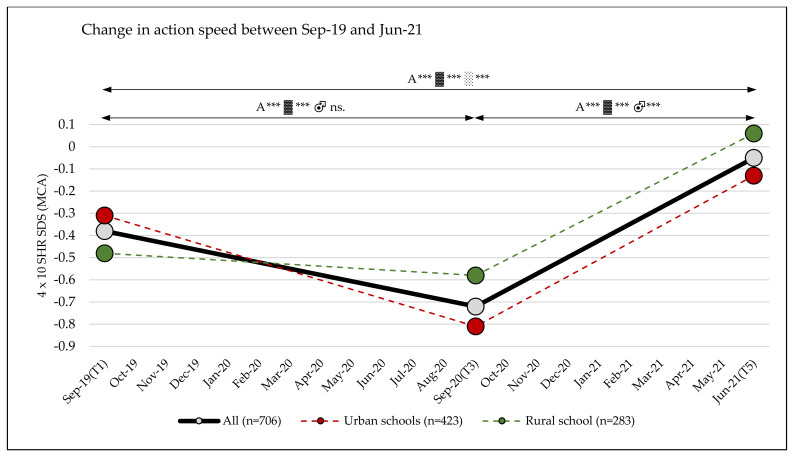
4 × 10 m shuttle run standard deviation scores over three test phases (T1, T3, T5); A = all, ▓ = urban, ░ = rural, 4 × 10 SHR = 4-m × 10-m shuttle run, SDS = standard deviation score; significance level *p*-value: *** = *p*-value ≤ 0.001, ns. = *p*-value not significant.

Significant changes with large effects over time were observed in action speed (4 × 10 SHR SDS (MCA)) (T1 to T5: main effect time: η_p_^2^ = 0.221; *p* < 0.001). A decrease in performance between September 2019 and September 2020 (−0.34 (95% CI, −0.41–−0.27)) was followed by a large increase in performance between September 2020 and June 2021 (+0.67 (95% CI, 0.60–0.73)). The decrease in performance was more pronounced in children attending urban schools (−0.51 (95% CI, −0.59–−0.42)) than in children attending rural schools (−0.10 (95% CI, −0.20–0.00)) (Table 3 and Table 4, and Table S8, Figure 7). No differences were found between subgroups for sex or school grade (Table 3).

### 3.4. Change in Weight Classifications

**Figure 8:**
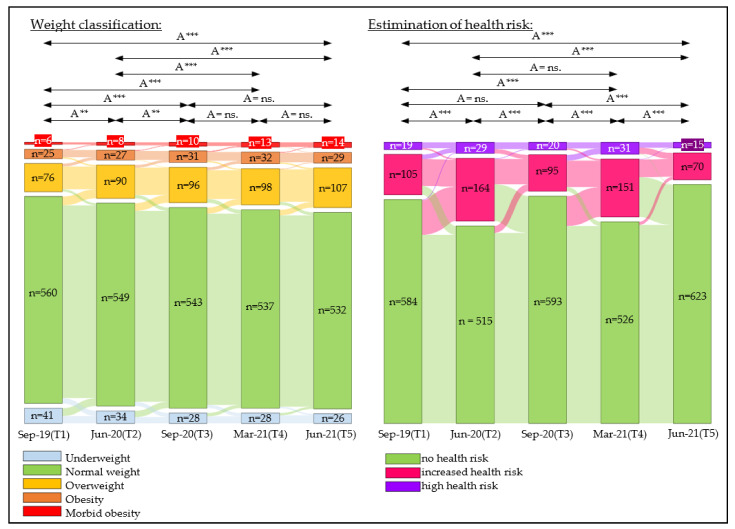
Changes in weight classification and estimation of health risk groups over five test phases (T1, T2, T3, T4, and T5); A = all, *n* = number of children; significance level *p*-value: ** = *p*-value ≤ 0.01, *** = *p*-value ≤ 0.001, ns. = *p*-value not significant.

During the observation period September 2019 to June 2021, there was a continuous increase in the number of children with overweight, obesity, or extreme obesity (September 2019: *n* = 107; June 2021: *n* = 150). This increase was more pronounced in boys and children attending rural schools than in girls and children attending urban schools (Table 2 and Tables S9 and S10, Figure 8).

### 3.5. Change in Estimation of Health Risk

The number of children with estimated increased or high health risk showed a strong increase after the first (September 2019 to June 2020 (*n*= 124 to 193)) and second lockdown phase (September 2020 to March 2021 (*n* = 115 to 182)), respectively, but decreased again significantly during the first (June 2020 to September 2020 (*n* = 193 to 115)) and second opening phase (March 2021 to June 2021 (*n* = 182 to 85)), respectively. In the first lockdown phase (September 2019 to June 2020), boys and children attending urban schools showed a larger increase in both classification groups of increased and high health risk than girls and children attending rural schools. In the second lockdown phase, the increase was equally large in all subgroups (sex and school location) (Table 2 and Tables S9 and S11, Figure 8).

### 3.6. Change in Classification of Cardiorespiratory Endurance

**Figure 9:**
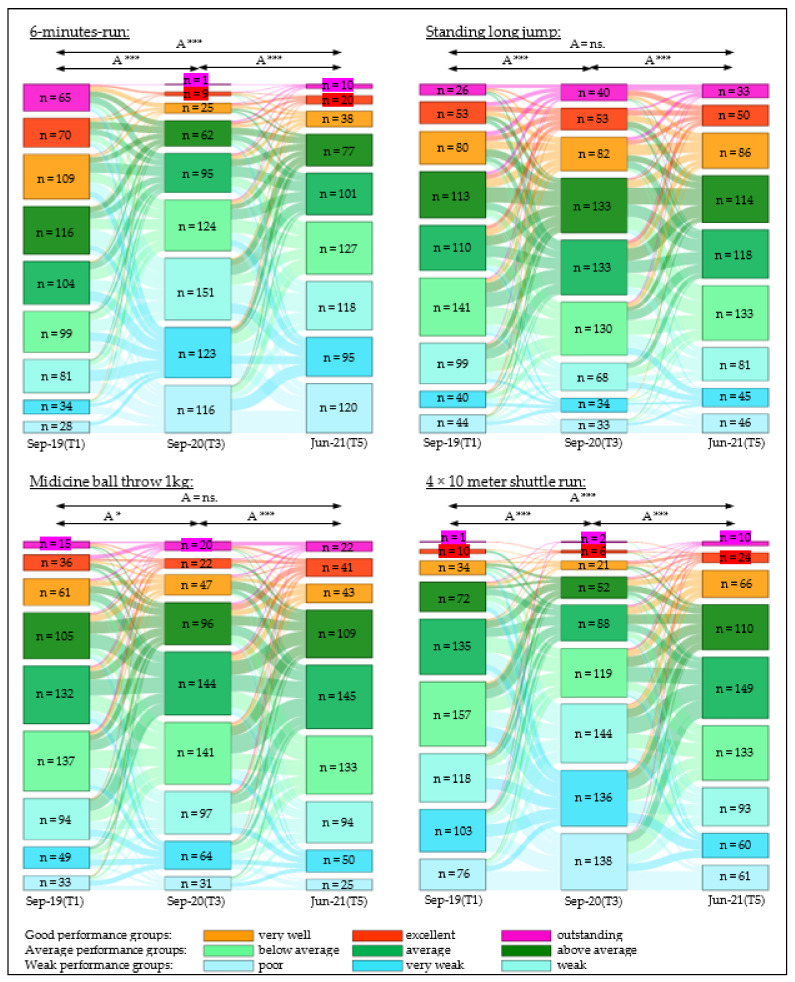
Changes in fitness performance groups over three test phases (T1, T3, and T5); A = all, *n* = number of children; significance level *p*-value: * = *p*-value ≤ 0.05, *** = *p*-value ≤ 0.001, ns. = *p*-value not significant.

In the below-average to poor performance classes, the number of children increased sharply between September 2020 and June 2021 (*p* < 0.001). During the first lockdown period (September 2019 to June 2021), the number of children in the poor and very weak performance categories increased fourfold, from 4.0% to 16.4% (*p* < 0.001) and 4.8% to 17.4% (*p* < 0.001), respectively. During the same period, the number of children in the “excellent” and “outstanding” performance categories decreased significantly, from 9.9% to 1.3% and from 9.2% to 0.1%, respectively. In the second lockdown phase from October 2020 to March 2021, the number of children in the above average and very good performance categories increased (*p* < 0.001), but the number was significantly below (*p* < 0.001) the values of the baseline measurement (Figure 9, Tables S12–S14).

### 3.7. Change in the Classification of Muscle Strength

There were no significant changes in the number of children in the different performance categories of the standing long jump or medicine ball throw (SLJ, *p* = 0.69; MB1 kg, *p* > 0.99) between the baseline measurement (September 2019) and the measurement at the end of the observation period (Jun-21) (Figure 9, Tables S12–S14).

### 3.8. Change in the Classification of Action Speed

After the first lockdown phase (September 2019 to September 2020), there was a significant decrease (*p* < 0.001) in the number of children in the better performance groups. From September 2020 onwards, a trend reversal took place at the end of the observation period (June 2021); the number of children in the average and better performance groups was higher (*p* < 0.001) than at the baseline measurement (September 2019) (Figure 9, Tables S12–S14).

## 4. Discussion

Our results showed an increase in mean BMI SDS values during the COVID-19 pandemic and, as a result, the number of children suffering from overweight, obesity, or extreme obesity in our sample increased from 15.0% to 21.2%, representing a relative increase of 41.3%. Waist circumference showed increases during lockdown phases and decreases after COVID-19 measures were relaxed. A dramatic loss of cardiorespiratory endurance and action speed between September 2019 and September 2020 was followed by a slight upward trend at the end of the observation period (June 2021). Muscle strength showed no changes during the COVID-19 pandemic.

In line with our findings, there are already a number of studies reporting an increase in BMI associated with the COVID-19 pandemic [37,44,59,60,61]. However, only a few studies [44,46] have objectively measured health-related fitness from a representative sample, and the abdominal circumference results described in our study are the first such presented worldwide.

Although changes in waist circumference are reported to be more useful than BMI changes for detecting behavioral changes related to changes in energy balance [62], waist circumference tends to vary with clock time and eating and drinking patterns, and is subject to seasonal variation [62,63]. Waist-to-height ratio is a better screening tool for cardiometabolic risk factors than BMI and waist circumference [64,65]. We found that waist-to-height ratio strongly increased after the first (September 2019 to June 2020) and second (September 2020 to March 2021) lockdown phases, resulting in a strong increase in the number of children at increased or high health risk. The increased waist-to-height ratio likely represents a change in the energy balance of children during the lockdown phases. After periods without a lockdown (June 2020 to September 2020 and March 2021 to June 2021), the waist-to-height ratio decreased again and, as a result, the number of children with an increased or high health risk decreased. This indicates that WHtR is a very responsive measure which showed clear negative effects on child health immediately related to COVID-19 mitigation measures and fortunately improved again after periods without mitigation measures.

Health-related fitness, particularly cardiorespiratory endurance (6MR), showed worrisome and dramatic effects associated with the COVID-19 era. Studies from the United States, Australia, and Europe have reported that between 1974 and 2015 fitness was relatively stable, with annual changes of around 0.10 SDS [19]. This makes the loss in cardiorespiratory fitness of more than one standard deviation (6MR SDS (DüMo) = −1.08) from September 2019 to September 2020 seen in our study even more alarming. Fortunately, some improvements were seen from September 2020 to June 2021, but cardiorespiratory fitness was not back to its original level. Specific measures for improving fitness are needed in the coming years to allow primary school children to recover the fitness that they lost during the COVID-19-related lockdown phases.

The results regarding the action speed showed a comparable pattern to that of cardiorespiratory endurance: a decrease during the first year of the pandemic was followed by an increase, even reaching a higher level at the end of the observation period when compared to the beginning. The reason for this surprising result might be a kind of “training effect”. When assessing the results of the 4-m × 10-m shuttle run, it should be considered that technique plays a major role in completing the turnaround points, and improved turnaround techniques can lead to a better overall performance. This could explain the significant increase in action speed between September 2020 and June 2021.

The slight changes found in the muscle strength tests (standing long jump and medicine ball throw) were marginal compared to the results of the 6 min run or 4 × 10 m shuttle run and did not show any significant changes between the beginning and the end of the observation period. This could be related to the fact that strength gains in this age group are usually not generated by specific strength training [66]. Therefore, COVID-19-related mitigation measures seem to have no long-term negative effects on children in terms of muscle strength.

A major strength of our study is that all data were collected by a team of six trained sports scientists and sports educators, and that individual measurements in the different test phases were carried out by the same test instructor. Another strength is that all the data collected were objectively measured. A further strength is the representative sample size, which allows for a general statement to be made about the development in fitness and health status of primary school children in Austria during the course of the COVID-19 pandemic. Furthermore, the existence of current national reference values (EQUI BMI) may be mentioned as an additional strength.

A limitation of this study is the lack of national reference values for fitness. An additional weakness related to the fitness reference values used is the fact that the medicine ball throw data were available only in yearly steps and this could have had an influence on the SDS values in the second observation period (September 2020 to June 2021 = time span of only nine months).

## 5. Conclusions

Our study shows that COVID-19-related mitigation measures had worrisome negative health effects on primary school-aged children in Austria. Our findings provide a basis for political decision makers concerning mitigation measures in the future. The advantage of these with respect to virus transmission has to be carefully balanced against the long-term disadvantages with respect to BMI and fitness and thus physical health in developing individuals, i.e., children and adolescents. Continued physical activity—that is also practiced in schools—should be considered similarly important to infection prevention.

To guarantee “safe schools” and to allow the maintenance of physical education in classes, daily rapid antigen testing during periods of high COVID-19 incidence could be an option. Close cooperation of school staff, physicians, governmental and health authorities, and other key players is needed to keep activity restrictions at a minimum and thus to allow the best possible health development for school children.

In the near future, politicians should implement adequate interventions (daily physical activity lessons, healthy snacks) to counteract the negative developments triggered by the COVID-19 pandemic. Furthermore, the long-term follow-up of children affected by COVID-19 mitigation measures is warranted to monitor their future health and fitness development. Therefore, it would also be important to follow the example of other countries [28,29] and implement a nationwide health monitoring system (e.g., AUT FIT [48]) in primary and secondary schools.

## Figures and Tables

**Table 1:** Overall sample characteristics.

Variable	September 2019	June 2020	September-2020	March 2021	June 2021
Age (years), mean (SD)	8.3 (0.7)	9.0 (0.7)	9.2 (0.7)	9.7 (0.7)	9.9 (0.7)
SY 19–20—grade 2, age (years), mean (SD)	7.7 (0.4)		8.7 (0.4)		9.3 (0.4)
SY 19–20—grade 3, age (years), mean (SD)	8.8 (0.5)		9.7 (0.5)		10.4 (0.5)
Female sex, No. (%)	350 (49.4%)				
Urban school, No. (%)	424 (59.9%)				
SY 19–20—grade 2, No. (%)	345 (48.7%)				

Study population, *n* = 708, data are No (%) or mean (SD). SY = school year.

**Table 2:** Overall sample characteristics of anthropometrics.

Variable	Subgroups/ Categories	September 2019	June 2020	September-2020	March 2021	June 2021
EQUI BMI_AUT_, mean (SD)	All (*n* = 708)	22.30 (3.51)	22.60 (3.76)	22.73 (3.85)	22.93 (3.97)	22.98 (3.95)
	Girls (*n* = 350)	22.12 (3.39)	22.28 (3.48)	22.39 (3.61)	22.43(3.65)	22.53 (3.56)
	Boys (*n* = 358)	22.47 (3.63)	22.91 (4.00)	23.06 (4.05)	23.42 (4.12)	23.41 (4.26)
	Urban schools (*n* = 424)	22.39 (3.70)	22.75 (4.11)	22.92 (4.16)	23.22 (4.34)	23.24 (4.30)
	Rural school (*n* = 284)	22.17 (3.22)	22.37 (3.16)	22.45 (3.32)	22.49 (3.33)	22.57 (3.32)
AUT weight classification, No. (%)—All (*n* = 708)	Underweight	41 (5.8)	34 (4.8)	28 (4.0)	28 (4.0)	26 (3.7)
	Normal weight	560 (79.1)	549 (77.5)	543 (76.7)	537 (75.8)	532 (75.1)
	Overweight	76 (10.7)	90 (12.7)	96 (13.6)	98 (13.8)	107 (15.1)
	Obesity	25 (3.5)	27 (3.8)	31 (4.4)	32 (4.5)	29 (4.1)
	Morbid obesity	6 (0.8)	8 (1.1)	10 (1.4)	13 (1.8)	14 (2.0)
WHtR, Mean (SD)	All (*n* = 708)	0.461 (0.052)	0.480 (0.058)	0.450 (0.057)	0.472 (0.060)	0.434 (0.057)
	Girls (*n* = 350)	0.464 (0.053)	0.480 (0.058)	0.449 (0.056)	0.469 (0.060)	0.429 (0.055)
	Boys (*n* = 358)	0.458 (0.050)	0.479 (0.058)	0.452 (0.058)	0.475 (0.061)	0.439 (0.059)
	Urban schools (*n* = 424)	0.455 (0.054)	0.482 (0.062)	0.453 (0.062)	0.476 (0.063)	0.438 (0.061)
	Rural school (*n* = 284)	0.469 (0.047)	0.475 (0.051)	0.446 (0.049)	0.467 (0.055)	0.429 (0.050)
WHtR, heath estimation, No. (%)—All (*n* = 708)	no health risk	584 (82.5)	515 (72.7)	593 (83.8)	526 (74.3)	623 (88.0)
	increased health risk	105 (14.8)	164 (23.2)	95 (13.4)	151 (21.3)	70 (9.9)
	high health risk	19 (2.7)	29 (4.1)	20 (2.8)	31 (4.4)	15 (2.1)

Data are No (%) or mean (SD); EQUI BMI_AUT_ = equivalent BMI based on Austrian reference centile curves passing through adult BMI values [53], AUT weight classification = based on Austrian reference centile curves passing through adult BMI values [53], weight classification = underweight = equivalent BMI < 18.5, normal weight = equivalent BMI = 18.5 to 25, overweight = equivalent BMI = 25.0 to 30.0, obesity = equivalent BMI = 30 to 35, morbid obesity = equivalent BMI > 35; WHtR = waist-to-height ratio, estimation of health risk: no health risk = WHtR < 0.5, increased health risk = WHtR 0.5 to < 0.6, high health risk = WHtR ≥ 0.6.

**Table 3:** Mixed ANOVAs for anthropometrics and fitness.

Variable	Effects	Time and Subgroups	df	F	*p*-Value	η_p_^2^	Power ^a^
EQUI BMI_AUT_	Between-subjects effects	Sex	1	5.824	0.016	0.008	0.674
		School Location	1	2.470	0.116	0.004	0.348
		School Grade	1	0.095	0.758	0.000	0.061
		Sex × School Location	1	0.061	0.805	0.000	0.057
		Sex × School Grade	1	1.054	0.305	0.002	0.176
		School Location × School Grade	1	1.290	0.256	0.002	0.205
		Sex × School Location × School Grade	1	2.459	0.117	0.004	0.347
		Error	700				
	Within-subjects effects	Time (T1–T2–T3–T4–T5)	2.868	67.098	0.000	0.087	1.000
		Time × Sex	2.868	15.483	0.000	0.022	1.000
		Time × School Location	2.868	8.941	0.000	0.013	0.995
		Time × School Grade	2.868	0.667	0.566	0.001	0.188
		Time × Sex × School Location	2.868	0.651	0.575	0.001	0.185
		Time × Sex × School Grade	2.868	2.743	0.044	0.004	0.653
		Time × School Location × School Grade	2.868	1.484	0.219	0.002	0.386
		Time × Sex × School Location × School Grade	2.868	0.398	0.746	0.001	0.128
		Error (Time)	2007.936				
WHtR	Between-subjects effects	Sex	1	0.424	0.515	0.001	0.100
		School Location	1	0.741	0.390	0.001	0.138
		School Grade	1	0.050	0.823	0.000	0.056
		Sex × School Location	1	0.055	0.814	0.000	0.056
		Sex × School Grade	1	1.505	0.220	0.002	0.232
		School Location × School Grade	1	2.256	0.134	0.003	0.323
		Sex × School Location × School Grade	1	0.968	0.326	0.001	0.166
		Error	700				
	Within-subjects effects	Time (T1-T2-T3-T4-T5)	3.848	384.997	0.000	0.355	1.000
		Time × Sex	3.848	9.706	0.000	0.014	1.000
		Time × School Location	3.848	26.781	0.000	0.037	1.000
		Time × School Grade	3.848	2.024	0.091	0.003	0.597
		Time × Sex × School Location	3.848	1.145	0.333	0.002	0.356
		Time × Sex × School Grade	3.848	0.912	0.453	0.001	0.287
		Time × School Location × School Grade	3.848	2.584	0.037	0.004	0.719
		Time × Sex × School Location × School Grade	3.848	0.383	0.814	0.001	0.137
		Error (Time)	2693.887				
6MR SDS (DüMo)	Between-subjects effects	Sex	1	6.731	0.010	0.010	0.736
		School Location	1	0.064	0.800	0.000	0.057
		School Grade	1	0.060	0.806	0.000	0.057
		Sex × School Location	1	1.408	0.236	0.002	0.220
		Sex × School Grade	1	2.469	0.117	0.004	0.348
		School Location × School Grade	1	3.430	0.064	0.005	0.456
		Sex × School Location × School Grade	1	0.023	0.880	0.000	0.053
		Error	698				
	Within-subjects effects	Time (T1-T3-T5)	1.974	548.891	0.000	0.440	1.000
		Time × Sex	1.974	3.415	0.034	0.005	0.639
		Time × School Location	1.974	2.059	0.129	0.003	0.422
		Time × School Grade	1.974	6.580	0.002	0.009	0.908
		Time × Sex × School Location	1.974	0.843	0.429	0.001	0.194
		Time × Sex × School Grade	1.974	1.661	0.191	0.002	0.349
		Time × School Location × School Grade	1.974	1.968	0.141	0.003	0.406
		Time × Sex × School Location × School Grade	1.974	0.009	0.990	0.000	0.051
		Error (Time)	1377.609				
SLJ SDS (DüMo)	Between-subjects effects	Sex	1	1.083	0.298	0.002	0.180
		School Location	1	1.581	0.209	0.002	0.241
		School Grade	1	0.229	0.633	0.000	0.077
		Sex × School Location	1	1.833	0.176	0.003	0.272
		Sex × School Grade	1	0.299	0.585	0.000	0.085
		School Location × School Grade	1	2.545	0.111	0.004	0.357
		Sex × School Location × School Grade	1	0.262	0.609	0.000	0.080
		Error	698				
	Within-subjects effects	Time (T1-T3-T5)	1.977	22.323	0.000	0.031	1.000
		Time × Sex	1.977	1.604	0.202	0.002	0.339
		Time × School Location	1.977	12.384	0.000	0.017	0.996
		Time × School Grade	1.977	34.908	0.000	0.048	1.000
		Time × Sex × School Location	1.977	2.359	0.096	0.003	0.476
		Time × Sex × School Grade	1.977	0.948	0.387	0.001	0.214
		Time × School Location × School Grade	1.977	0.036	0.964	0.000	0.055
		Time × Sex × School Location × School Grade	1.977	0.753	0.470	0.001	0.178
		Error (Time)	1379.618				
MB1 kg z-value (KATS-K)	Between-subjects effects	Sex	1	12.770	0.000	0.019	0.946
		School Location	1	0.590	0.443	0.001	0.120
		School Grade	1	0.208	0.648	0.000	0.074
		Sex × School Location	1	2.105	0.147	0.003	0.305
		Sex × School Grade	1	0.799	0.372	0.001	0.145
		School Location × School Grade	1	0.441	0.507	0.001	0.102
		Sex × School Location × School Grade	1	0.618	0.432	0.001	0.123
		Error	654				
	Within-subjects effects	Time (T1–T3–T5)	1.948	6.966	0.001	0.011	0.921
		Time × Sex	1.948	8.643	0.000	0.013	0.966
		Time × School Location	1.948	3.429	0.034	0.005	0.637
		Time × School Grade	1.948	5.551	0.004	0.008	0.848
		Time × Sex × School Location	1.948	0.185	0.826	0.000	0.078
		Time × Sex × School Grade	1.948	0.823	0.436	0.001	0.190
		Time × School Location × School Grade	1.948	2.783	0.064	0.004	0.542
		Time × Sex × School Location × School Grade	1.948	6.530	0.002	0.010	0.903
		Error (Time)	1273.678				
4 × 10 SHR SDS (MCA), mean (SD)	Between-subjects effects	Sex	1	1.691	0.194	0.002	0.255
		School Location	1	2.026	0.155	0.003	0.295
		School Grade	1	1.325	0.250	0.002	0.210
		Sex × School Location	1	1.404	0.236	0.002	0.220
		Sex × School Grade	1	0.969	0.325	0.001	0.166
		School Location × School Grade	1	1.001	0.317	0.001	0.170
		Sex × School Location × School Grade	1	1.108	0.293	0.002	0.183
		Error	698				
	Within-subjects effects	Time (T1–T3–T5)	2	198.380	0.000	0.221	1.000
		Time × Sex	2	3.929	0.020	0.006	0.708
		Time × School Location	2	20.824	0.000	0.029	1.000
		Time × School Grade	2	3.488	0.031	0.005	0.653
		Time × Sex × School Location	2	3.650	0.026	0.005	0.674
		Time × Sex × School Grade	2	1.013	0.364	0.001	0.228
		Time × School Location × School Grade	2	2.893	0.056	0.004	0.567
		Time × Sex × School Location × School Grade	2	1.242	0.289	0.002	0.272
		Error (Time)	1396				

^a^ Observed power computed using alpha = 0.05. ANOVA = analysis of variance, BMI = body mass index, df = degrees of freedom, F = test statistic, $\eta_{p}^{2}$ = partial eta square, EQUI BMI_AUT_ = equivalent BMI based on Austrian reference centile curves passing through adult BMI values [53], AUT weight classification = based on Austrian reference centile curves passing through adult BMI values [53], WHtR = waist-to-height ratio, SD = standard deviation; 6 MR = 6 min run, SLJ = standing long jump, MB1 kg = medicine ball throw (1 kg), 4 × 10 SHR = 4 × 10 m shuttle run, SDS = standard deviation score, z-value = traditional z-score standardization; DüMo = Düsseldorfer model [28], KATS-K = Karlsruher test system [57], MCA = motor competence assessment [55].

**Table 4:** Overall sample characteristics of fitness.

Fitness Test	Subgroup/Categories	September 2019	September 2020	June 2021
6MR SDS (DüMo), mean (SD)	All (*n* = 706)	0.49 (1.12)	−0.59 (0.93)	−0.41 (1.08)
	Girls (*n* = 349)	0.42 (1.05)	−0.65 (0.88)	−0.57 (1.01)
	Boys (*n* = 357)	0.57 (1.18)	−0.52 (0.98)	−0.25 (1.13)
	Urban schools (*n* = 423)	0.48 (1.13)	−0.58 (1.02)	−0.37 (1.14)
	Rural school (*n* = 283)	0.52 (1.11)	−0.60 (0.80)	−0.48 (0.99)
	SY 19–20—grade 2 (*n* = 345)	0.44 (1.10)	−0.53 (0.92)	−0.33 (1.11)
	SY 19–20—grade 3 (*n* = 361)	0.55 (1.14)	−0.63 (0.92)	−0.50 (1.06)
SLJ SDS (DüMo), mean (SD)	All (*n* = 706)	0.18 (1.06)	0.35 (1.05)	0.23 (1.07)
	Girls (*n* = 349)	0.10 (1.02)	0.33 (1.02)	0.18 (1.03)
	Boys (*n* = 357)	0.25 (1.09)	0.37 (1.08)	0.28 (1.10)
	Urban schools (*n* = 423)	0.21 (1.05)	0.25 (1.08)	0.20 (1.08)
	Rural school (*n* = 283)	0.13 (1.07)	0.50 (0.98)	0.29 (1.08)
	SY 19–20—grade 2 (*n* = 345)	0.06 (1.00)	0.44 (1.06)	0.37 (1.07)
	SY 19–20—grade 3 (*n* = 361)	0.29 (1.10)	0.27 (1.04)	0.10 (1.05)
MB1 kg z-value (KATS-K), mean (SD)	All (*n* = 662)	0.11 (0.95)	0.02 (0.93)	0.14 (0.97)
	Girls (*n* = 330)	0.14 (0.93)	0.19 (0.91)	0.25 (0.93)
	Boys (*n* = 332)	0.07 (0.97)	−0.14 (0.93)	0.03 (0.99)
	Urban schools (*n* = 394)	0.05 (0.98)	−0.01 (0.94)	0.14 (1.00)
	Rural school (*n* = 268)	0.19 (0.89)	0.07 (0.92)	0.13 (0.93)
	SY 19–20—grade 2 (*n* = 344)	0.11 (0.97)	0.11 (0.93)	0.10 (0.97)
	SY 19–20—grade 3 (*n* = 318)	0.10 (0.93)	−0.07 (0.93)	0.18 (0.96)
4 × 10 SHR SDS (MAC), mean (SD)	All (*n* = 706)	−0.38 (0.92)	−0.72 (1.00)	−0.05 (1.01)
	Girls (*n* = 349)	−0.47 (0.93)	−0.78 (0.96)	−0.05 (0.95)
	Boys (*n* = 357)	−0.29 (0.90)	−0.66 (1.04)	−0.06 (1.06)
	Urban schools (*n* = 423)	−0.31 (0.90)	−0.81 (1.02)	−0.13 (1.06)
	Rural school (*n* = 283)	−0.48 (0.94)	−0.58 (0.95)	0.06 (0.91)
	SY 19–20—grade 2 (*n* = 345)	−0.38 (0.88)	−0.62 (0.98)	−0.00 (0.96)
	SY 19–20—grade 3 (*n* = 361)	−0.37 (0.96)	−0.81 (1.01)	−0.11 (1.04)

Data are mean (SD); SD = standard deviation; 6MR = 6 min run, SLJ = standing long jump, MB1 kg = medicine ball throw (1 kg), 4 × 10 SHR = 4-m × 10-m shuttle run, SDS = standard deviation score, z-value = traditional z-score standardization; DüMo = Düsseldorfer model [28], KATS-K = Karlsruher test system [57], MCA = motor competence assessment [55], SY 19–20—grade 2 = children in grade 2 in the 2019/20 school year grade, SY 19–20—grade 3 = children in grade 3 of the 2019/20 school year.

## Data Availability

The data presented in this study are available on request from the corresponding author. The data are not publicly available due to privacy/ethical restrictions.

## Supplementary Materials

The following are available online at https://www.mdpi.com/article/10.3390/sports10030043/s1: Table S1: Restriction levels for children in Austria from 31 January 2020 to 30 June 2021 in relation to the OxCGRT stringency index, Table S2: Detailed description of the restrictions for Austrian children in primary school from 31 January 2020 to 30 June 2021 in relation to the OxCGRT stringency index, Table S3: Overall sample characteristics study population vs. loss at follow-up, Table S4: Additional sample characteristics for anthropometrics for subgroups of sex and school location, Table S5: Additional sample characteristics for fitness tests for subgroups of sex, school location, and school grade, Table S6: Post hoc tests for EQUI BMI AUT for the main effect time and interactions for time × sex and time × school location based on the estimated marginal means, Table S7: Post hoc tests for waist-to-height ratio for the main effect time and interactions for time × sex and time × school location based on the estimated marginal means, Table S8: Post hoc tests for fitness tests for the main effect time and interactions for time × sex, time × school location and time × school grade based on the estimated marginal means, Table S9: Overview of fitness test STA9 classifications in relation to the results of international reference values, Table S10: Friedman test for weight classification and estimation of health risk using cut-offs described in AUT FIT to baseline measurements T1 and follow-up measurements T2, T3, T4, and T5, Table S11: Friedman test for fitness performance categories using cut-offs described in AUT FIT for baseline measurements T1 and follow-up measurements T3 and T5, Table S12: Post hoc analyses by the Wilcoxon test for weight classifications using Austrian cut-offs, Table S13: Post hoc analyses by the Wilcoxon test for the estimation of health risk using cut-offs described in AUT FIT, Table S14: Post hoc analyses by the Wilcoxon test for the estimation of health risk using cut-offs described in AUT FIT, Figure S1: COVID-19 restrictions in Austria between 31 January 2020 and 30 June 2021.
