# Supplementary material for: The Impact of COVID-19-Related Mitigation Measures on the Health and Fitness Status of Primary School Children in Austria: A Longitudinal Study with Data from 708 Children Measured before and during the Ongoing COVID-19 Pandemic
Source: Sports (Basel). 2022 Mar 11;10(3):43. doi: 10.3390/sports10030043 (PMC8949892; doi:10.3390/sports10030043)
Supplement: Supplementary file 1 [file sports-10-00043-s001.zip › sports-1559641-supplementary.pdf]

The impact of COVID-19 related mitigation measures on the health and fitness status of primary school children in Austria: A longitudinal study with data from 708 children measured before and during the ongoing COVID-19 pandemic.

Supplementary material

Gerald Jarnig, Reinhold Kerbl and Mireille N.M. van Poppel

This supplementary material has been provided by the authors to give readers additional information about their work.

Table S1. Restriction levels for children in Austria from January 31, 2020 to June 30, 2021 in relation to the OxCGRT stringency index.

Table S2. Detailed description of the restrictions for Austrian children in primary school from January 31, 2020 to June 30, 2021 in relation to the OxCGRT stringency index.

Table S3. Overall sample characteristics study population vs. loss at follow-up.

Table S4. Additional sample characteristics for anthropometrics for subgroups sex and school location.

Table S5. Additional sample characteristics for fitness tests for subgroups sex, school location and school grade.

Table S6. Post hoc tests for EQUI BMI_{AUT} for the main effect time and interactions for time × sex and time × school location based on the estimated marginal means.

Table S7. Post hoc tests for waist-to-height ratio for the main effect time and interactions for time × sex and time × school location based on the estimated marginal means.

Table S8. Post hoc tests for fitness tests for the main effect time and interactions for time × sex, time × school location, and time × school grade based on the estimated marginal means.

Table S9. Friedman test for weight classification and estimation of health risk using cut-offs described in AUT FIT to baseline measurements T1 and follow-up measurements T2, T3, T4, and T5.

Table S10. Post hoc analyses by the Wilcoxon test for weight classifications using Austrian cut-offs.

Table S11. Post hoc analyses by the Wilcoxon test for estimation of health risk using cut-offs described in AUT FIT.

Table S12. Overview of fitness test STA9 classifications in relation to the results of international reference values.

Table S13. Friedman test for fitness performance categories using cut-offs described in AUT FIT in relation to baseline measurements T1 and follow-up measurements T3 and T5.

Table S14. Post hoc analyses by the Wilcoxon test for fitness tests using cut-offs described in AUT FIT.

Figure S1. COVID-19 restrictions in Austria between January 31, 2020 and June 30, 2021.

Figure S1A. Restriction levels for primary school children.

Figure S1B. OxCGRT Stringency Index.

Table S1. Restriction levels for children in Austria from January 31, 2020 to June 30, 2021 in relation to the OxCGRT Stringency Index.

Period	OxCGRT Austria	School Structure	Physical Education In school	Sports and Leisure Facilities	Sport at the Club	Stay at Home Requirements	Sum of Restrictions in the School
01-31-2020 – 02-23-2020	0.00	0	0	0	0	0	0
02-24-2020 – 03-08-2020	11.11	0	0	0	0	0	0
03-09-2020 – 03-11-2020	19.44	0	0	0	0	0	0
03-12-2020	34.26	0	0	0	0	0	0
03-13-2020 – 03-15-2020	48.15	0	0	0	0	0	0
03-16-2020 – 04-13-2020	81.48	3	3	3	3	3	15
04-14-2020 – 04-22-2020	77.78	3	3	3	3	3	15
04-23-2020 – 04-30-2020	75.00	3	3	3	3	3	15
05-01-2020	67.59	3	3	2	3	2	13
05-02-2020 – 05-10-2020	64.81	3	3	2	3	2	13
05-11-2020 – 05-17-2020	59.26	3	3	2	3	2	13
05-18-2020 – 05-28-2020	59.26	2	3	2	3	2	12
05-29-2020 – 06-02-2020	53.70	2	3	2	2	2	11
06-03-2020	50.00	2	3	2	2	2	11
06-04-2020 – 07-01-2020	47.22	2	3	2	2	2	11
07-02-2020 – 07-09-2020	47.22	2	3	0	1	0	6
07-10-2020 – 09-05-2020	35.19	0	0	0	1	0	1
09-06-2020 – 09-13-2020	36.11	0	0	0	1	0	1
09-14-2020 – 09-16-2020	36.11	1	0	0	1	1	3
09-17-2020 – 09-28-2020	37.04	1	0	0	1	1	3
09-29-2020 – 10-12-2020	40.74	1	0	0	1	1	3
10-13-2020 – 10-16-2020	44.91	1	0	0	1	2	4
10-17-2020 – 10-22-2020	58.80	1	0	0	1	2	4
10-23-2020	60.19	1	0	0	1	2	4
10-24-2020 – 10-26-2020	60.19	0	0	1	3	2	6
10-27-2020 – 11-01-2020	64.81	0	0	1	3	2	6
11-02-2020	75.00	0	0	1	3	2	6
11-03-2020 – 11-16-2020	75.00	0	2	1	3	2	8
11-17-2020 – 12-06-2020	82.41	3	3	1	3	2	12
12-07-2020 – 12-23-2020	71.30	1	2	1	3	1	8
12-24-2020 – 12-25-2020	82.41	3	3	1	3	2	12
12-26-2020 – 01-06-2021	82.41	3	3	1	3	2	12
01-07-2021 – 01-18-2021	82.41	3	3	1	3	2	12
01-19-2021 – 02-05-2021	82.41	2	2	1	3	1	9
02-06-2021 – 02-07-2021	82.41	3	3	1	3	1	11
02-08-2021 – 02-11-2021	74.07	3	3	1	3	1	11
02-12-2021 – 02-14-2021	79.63	3	3	1	3	1	11
02-15-2021 – 03-14-2021	75.93	1	2	1	3	1	8
03-15-2021 – 03-31-2021	73.15	1	2	1	2	1	7

04-01-2021 – 04-05-2021	75.00	1	2	1	2	1	7
04-06-2021 – 04-25-2021	78.70	1	1	1	2	1	6
04-26-2021 – 05-02-2021	76.85	1	1	1	2	1	6
05-03-2021 – 05-10-2021	75.00	1	1	1	2	1	6
05-11-2021 – 05-15-2021	73.15	1	1	1	2	1	6
05-16-2021 – 05-18-2021	65.74	1	1	1	2	1	6
05-19-2021 – 06-09-2021	68.52	1	0	0	1	0	2
06-10-2021 – 06-30-2021	60.19	1	0	0	1	0	2

Level 0 = no restrictions, Level 1 = low restrictions, Level 2 = medium restrictions, Level 3 = high restrictions, OxCGRT = Oxford COVID-19 Government Response Tracker Stringency Index.

Table S2. Detailed description of the restrictions for Austrian children in primary school from January 31, 2020 to June 30, 2021 in relation to the OxCGRT Stringency Index.

Period	OxCGRT Austria	School structure	Physical education in school	Sports and leisure facilities	Sport at the club	Stay at home requirements
01-31-2020 – 02-23-2020	0.00	A	A	A	A	A
02-24-2020 – 03-08-2020	11.11	A	A	A	A	A
03-09-2020 – 03-11-2020	19.44	A	A	A	A	A
03-12-2020	34.26	A	A	A	A	A
03-13-2020 – 03-15-2020	48.15	A	A	A	A	A
03-16-2020 – 04-13-2020	81.48	B	D	E	G	I
04-14-2020 – 04-22-2020	77.78	B	D	E	G	I
04-23-2020 – 04-30-2020	75.00	B	D	E	G	I
05-01-2020	67.59	B	D	F	G	J
05-02-2020 – 05-10-2020	64.81	B	D	F	G	J
05-11-2020 – 05-17-2020	59.26	B	D	F	G	J
05-18-2020 – 05-28-2020	59.26	C	D	F	G	J
05-29-2020 – 06-02-2020	53.70	C	D	F	H	J
06-03-2020	50.00	C	D	F	H	J
06-04-2020 – 07-01-2020	47.22	C	D	F	H	J
07-02-2020 – 07-09-2020	47.22	C	D	A	L	A
07-10-2020 – 09-05-2020	35.19	K	K	A	L	A
09-06-2020 – 09-13-2020	36.11	K	K	A	L	A
09-14-2020 – 09-16-2020	36.11	M	A	A	L	J
09-17-2020 – 09-28-2020	37.04	M	A	A	L	J
09-29-2020 – 10-12-2020	40.74	M	A	A	L	J
10-13-2020 – 10-16-2020	44.91	M	A	A	L	O
10-17-2020 – 10-22-2020	58.80	M	A	A	L	O
10-23-2020	60.19	M	A	A	L	O
10-24-2020 – 10-26-2020	60.19	K	K	F	G	O
10-27-2020 – 11-01-2020	64.81	K	K	F	G	O
11-02-2020	75.00	K	K	F	G	O
11-03-2020 – 11-16-2020	75.00	M	N	F	G	O
11-17-2020 – 12-06-2020	82.41	B	D	F	G	O
12-07-2020 – 12-23-2020	71.30	M	N	F	G	J
12-24-2020 – 12-25-2020	82.41	K	K	F	G	O
12-26-2020 – 01-06-2021	82.41	K	K	F	G	O
01-07-2021 – 01-18-2021	82.41	B	D	F	G	O
01-19-2021 – 02-05-2021	82.41	C	N	F	G	J

02-06-2021 – 02-07-2021	82.41	K	K	F	G	J
02-08-2021 – 02-11-2021	74.07	K	K	F	G	J
02-12-2021 – 02-14-2021	79.63	K	K	F	G	J
02-15-2021 – 03-14-2021	75.93	M	N	F	G	J
03-15-2021 – 03-31-2021	73.15	M	N	F	H	J
04-01-2021 – 04-05-2021	75.00	M	N	F	H	J
04-06-2021 – 04-25-2021	78.70	M	N	F	H	J
04-26-2021 – 05-02-2021	76.85	M	N	F	H	J
05-03-2021 – 05-10-2021	75.00	M	N	F	H	J
05-11-2021 – 05-15-2021	73.15	M	N	F	H	J
05-16-2021 – 05-18-2021	65.74	M	N	F	H	J
05-19-2021 – 06-09-2021	68.52	M	A	A	L	A
06-10-2021 – 06-30-2021	60.19	M	A	A	L	A

OxCGRT = Oxford COVID-19 Government Response Tracker Stringency Index, A = no restrictions, B = distance learning in primary school, C = lessons in primary school in shifts/home schooling are possible and recommended, D = no physical education in school, E = closures of all public playgrounds and sports facilities, F = public playgrounds and sports grounds are reopened, minimum distance must be maintained, G = club sport is forbidden, H = club sport is possible under severe restrictions and conditions, I = required with exceptions, J = restricted freedom of movement/keeping a minimum distance; K = holidays, L = practice of club sports is allowed under submission of prevention concepts, M = ordinary lessons in primary school/home schooling possible, N = limited sports activities in elementary school, O = recommended to stay at home.

Table S3. Overall sample characteristics study population vs. loss at follow-up.

Variable	Study population	loss at follow-up	X^2	t	p-lvl	*p*-Value
**Age (years)**	8.3 (0.7)	8.3 (0.7)		1.725		0.09
**Female Sex**	349 (49.4%)	69 (60.0%)	4.418		*	0.036
**Urban region**	423 (59.9%)	61 (53.0%)	1.914			0.17
**SY 19-20—grade 2**	344 (48.7%)	67 (58.3%)	3.596			0.06
**EQUI BMI_{AUT}**	22.30 (3.51)	21.79 (3.27)		1.449		0.15
**WHtR**	0.461 (0.520)	0.456 (0.48)		0.965		0.34
**6MR SDS (DüMo)**	0.49 (1.12)	0.24 (1.13)		2.252	*	0.025
**SLJ SDS (DüMo)**	0.18 (1.06)	−0.01 (1.11)		1.726		0.09
**MB1kg z-value (KATS-K)**	0.07 (0.95)	−0.12 (1.21)		1.561[a]		0.12[a]
**4-m × 10-m SHR SDS (MCA)**	−0.38 (0.92)	−0.70 (1.01)		3.476	**	0.001

Data are *n* (%) or mean (SD); sample size for study population, *n* = 708 for anthropometrics and *n* = 706 for motor fitness; for loss at follow up, *n* = 115 for anthropometrics and *n* = 117 for motor fitness, a = equal variances not assumed, X^2 = chi-square test value; t = test statistic *t*-test, p-lvl (*p*-value level) * = $p < 0.05$, ** = $p < 0.01$, *** = $p < 0.001$; BMI = body mass index, SY 19-20—grade 2 = children visiting in school year 2019/20 grade 2 of primary school, EQUI BMI_{AUT} = equivalent BMI based on Austrian reference centile curves passing through adult BMI values [1] , AUT weight classification = based on Austrian reference centile curves passing through adult BMI values [1], WHtR = waist-to-height ratio, SD = standard deviation; 6MR = 6 minute run, SLJ = standing long jump, MB1kg = medicine ball throw (1 kilogram), 4 x 10 SHR = 4-m x 10-m shuttle run, m = meter, SDS = standard deviation score, z-value = traditional z-score standardization; DüMo = Düsseldorfer Modell [2], KATS-K = Karlsruher test system [3], MCA = Motor Competence Assessment [4].

Table S4. Additional sample characteristics for anthropometrics for subgroups' sex and school location.

Variable	Subgroup	Categories	Sep-19	Jun-20	Sep-20	Mar-21	Jun-21
**AUT weight classification, no. (%)**	Girls	Underweight	20 (5.7)	19 (5.4)	20 (5.7)	22 (6.3)	22 (6.3)
		Normal weight	279 (79.7)	269 (76.9)	265 (75.7)	262 (74.9)	255 (72.9)
		Overweight	38 (10.9)	51 (14.6)	49 (14.0)	50 (14.3)	58 (16.6)
		Obesity	11 (3.1)	8 (2.3)	13 (3.7)	13 (3.7)	11 (3.1)
		Morbid obesity	2 (0.6)	3 (0.9)	3 (0.9)	3 (0.9)	4 (1.1)
	Boys	Underweight	21 (5.9)	15 (4.2)	8 (2.2)	6 (1.7)	4 (1.1)
		Normal weight	281 (78.5)	280 (78.2)	278 (77.7)	275 (76.8)	277 (77.4)
		Overweight	38 (10.6)	39 (10.9)	47 (13.1)	48 (13.4)	49 (13.7)
		Obesity	14 (3.9)	19 (5.3)	18 (5.0)	19 (5.3)	18 (5.0)
		Morbid obesity	4 (1.1)	5 (1.4)	7 (2.0)	10 (2.8)	10 (2.8)
	Urban schools	Underweight	25 (5.9)	22 (5.2)	15 (3.5)	15 (3.5)	14 (3.3)
		Normal weight	332 (78.3)	320 (75.5)	320 (75.5)	312 (73.6)	309 (72.9)
		Overweight	46 (10.8)	55 (13.0)	57 (13.4)	66 (15.6)	69 (16.3)
		Obesity	16 (3.8)	20 (4.7)	24 (5.7)	20 (4.7)	20 (4.7)
		Morbid obesity	5 (1.2)	7 (1.7)	8 (1.9)	11 (2.6)	12 (2.8)
	Rural schools	Underweight	16 (5.6)	12 (4.2)	13 (4.6)	13 (4.6)	12 (4.2)
		Normal weight	228 (80.3)	229 (80.6)	223 (78.5)	225 (79.2)	223 (78.5)
		Overweight	30 (10.6)	35 (12.3)	39 (13.7)	32 (11.3)	38 (13.4)
		Obesity	9 (3.2)	7 (2.5)	7 (2.5)	12 (4.2)	9 (3.2)
		Morbid obesity	1 (0.4)	1 (0.4)	2 (0.7)	2 (0.7)	2 (0.7)
**WHtR, heath estimation, no. (%)**	Girls	No h.r.	271 (77.4)	249 (71.1)	291 (83.1)	260 (74.3)	308 (88.0)
		Increased h.r.	72 (20.6)	88 (25.1)	53 (15.1)	77 (22.0)	39 (11.1)
		High h.r.	7 (2.0)	13 (3.7)	6 (1.7)	13 (3.7)	3 (0.9)
	Boys	No h.r.	313 (87.4)	266 (74.3)	302 (84.4)	266 (74.3)	315 (88.0)
		Increased h.r.	33 (9.2)	76 (21.2)	42 (11.7)	74 (20.7)	31 (8.7)
		High h.r.	12 (3.4)	16 (4.5)	14 (3.9)	18 (5.0)	12 (3.4)
	Urban schools	No h.r.	358 (84.4)	300 (70.8)	351 (82.8)	301 (73.1)	363 (85.6)
		Increased h.r.	53 (12.5)	101 (23.8)	56 (13.2)	90 (21.2)	49 (11.6)
		High h.r.	13 (3.1)	23 (5.4)	17 (4.0)	24 (5.7)	12 (2.8)
	Rural schools	No h.r.	226 (79.6)	215 (75.7)	242 (85.2)	216 (76.1)	260 (91.5)
		Increased h.r.	52 (18.3)	63 (22.2)	39 (13.7)	61 (21.5)	21 (7.4)
		High h.r.	6 (2.1)	6 (2.1)	3 (1.1)	7 (2.5)	3 (1.1)

Data are No (%); EQUI BMI_{AUT} = equivalent BMI based on Austrian reference centile curves passing through adult BMI values [1], AUT weight classification = based on Austrian reference centile curves passing through adult BMI values [1], Weight classification = Underweight = equivalent BMI = < 18.5, Normal weight = equivalent BMI = 18,5 to 25, Overweight = equivalent BMI = 25.0 to 30.0, Obesity = equivalent BMI = 30 to 35, Morbid obesity = equivalent BMI > 35; WHtR = waist-to-height ratio, h.r. = health risk, estimation of health risk: no health risk = WHtR < 0.5, increased health risk = WHtR 0.5 to < 0.6, high health risk = WHtR ≥ 0.6.

Table S5. Additional sample characteristics for Fitness tests for subgroups' sex, school location, and school grade.

Variable	Subgroup	Sep-19	Sep-20	Jun-21
**6MR, mean (SD), m**	All (*n* = 706)	918 (139)	814 (131)	861 (155)
	Girls (*n* = 349)	874 (118)	776 (111)	807 (128)
	Boys (*n* = 357)	960 (146)	850 (139)	913 (162)
	Urban schools (*n* = 423)	918 (141)	816 (141)	868 (162)
	Rural school (*n* = 283)	916 (137)	810 (115)	849 (145)
	SY 19-20—grade 2 (*n* = 345)	900 (134)	801 (126)	848 (153)
	SY 19-20—grade 3 (*n* = 361)	934 (142)	826 (135)	873 (157)
**SLJ, mean (SD), cm**	All (*n* = 706)	125 (20)	135 (20)	138 (21)
	Girls (*n* = 349)	119 (18)	130 (19)	133 (20)
	Boys (*n* = 357)	130 (20)	139 (20)	143 (22)
	Urban schools (*n* = 423)	125 (20)	134 (20)	138 (21)
	Rural school (*n* = 283)	123 (21)	137 (19)	139 (21)
	SY 19-20—grade 2 (*n* = 345)	120 (18)	132 (19)	136 (20)
	SY 19-20—grade 3 (*n* = 361)	129 (21)	138 (20)	141 (22)
**MB1kg, mean (SD), cm**	All (*n* = 706)	346 (72)	399 (73)	438 (78)
	Girls (*n* = 349)	316 (59)	374 (66)	411 (71)
	Boys (*n* = 357)	375 (72)	423 (71)	464 (76)
	Urban schools (*n* = 423)	346 (74)	401 (73)	443 (80)
	Rural school (*n* = 283)	346 (69)	396 (72)	431 (74)
	SY 19-20—grade 2 (*n* = 345)	318 (64)	376 (67)	414 (72)
	SY 19-20—grade 3 (*n* = 361)	373 (72)	421 (71)	462 (76)
**4 x 10 SHR, mean (SD), sec.**	All (*n* = 706)	14.90 (1.49)	14.76 (1.53)	13.49 (1.24)
	Girls (*n* = 349)	15.25 (1.47)	15.02 (1.42)	13.70 (1.17)
	Boys (*n* = 357)	14.57 (1.43)	14.51 (1.60)	13.29 (1.27)
	Urban schools (*n* = 423)	14.77 (1.42)	14.88 (1.57)	13.58 (1.32)
	Rural school (*n* = 283)	15.11 (1.57)	14.58 (1.45)	13.37 (1.09)
	SY 19-20—grade 2 (*n* = 345)	15.28 (1.43)	14.95 (1.53)	13.67 (1.18)
	SY 19-20—grade 3 (*n* = 361)	14.55 (1.45)	14.58 (1.51)	13.32 (1.27)

Data are mean (SD); n = sample size for study population, SD = standard deviation; 6MR = 6 minute run, SLJ = standing long jump, MB1kg = medicine ball throw (1 kilogram), 4 x 10 SHR = 4 x 10-meter shuttle run, m = meter, cm = centimeter, sec. = seconds, SY 19-20—grade 2 = children visiting in school year 2019/20 grade 2 of primary school, SY 19-20—grade 3 = children visiting in school year 2019/20 grade 3 of primary school.

Table S6. Post hoc tests for EQUI BMIAUT for the main effect time and interactions for time × sex and time × school location based on the estimated marginal means.

Variable	Subgroups	Pairwise comparisons	Mean diff (95% CI)	SE	p-lvl	*p*-Value[a]
EQUI BMIAUT	Time	T1 to T2	−0.283 (−0.409 to −0.157)	0.045	***	<0.001
		T1 to T3	−0.403 (−0.537 to −0.268)	0.048	***	<0.001
		T1 to T4	−0.583 (−0.733 to −0.433)	0.053	***	<0.001
		T1 to T5	−0.636 (−0.793 to −0.480)	0.056	***	<0.001
		T2 to T3	−0.120 (−0.228 to −0.012)	0.038	***	<0.001
		T2 to T4	−0.300 (−0.424 to −0.177)	0.044	***	<0.001
		T2 to T5	−0.353 (−0.486 to −0.220)	0.047	***	<0.001
		T3 to T4	−0.181 (−0.280 to −0.081)	0.035	***	<0.001
		T3 to T5	−0.234 (−0.348 to −0.120)	0.041	***	<0.001
		T4 to T5	−0.053 (−0.128 to −0.021)	0.027		0.45
	Time × Sex (Girls)	T1 to T2	−0.140 (−0.317 to 0.037)	0.063		0.26
		T1 to T3	−0.247 (−0.436 to −0.058)	0.067	**	0.003
		T1 to T4	−0.269 (−0.480 to −0.059)	0.075	**	0.003
		T1 to T5	−0.366 (−0.586 to −0.147)	0.078	***	<0.001
		T2 to T3	−0.107 (−0.258 to −0.045)	0.054		0.48
		T2 to T4	−0.129 (−0.302 to −0.044)	0.061		0.36
		T2 to T5	-0.226 (−0.412 to −0.040)	0.066	**	0.007
		T3 to T4	−0.023 (−0.162 to 0.117)	0.049		>0.99
		T3 to T5	−0.119 (−0.279 to 0.041)	0.057		0.36
		T4 to T5	−0.097 (−0.201 to 0.008)	0.037		0.10
	Time × Sex (Boys)	T1 to T2	−0.425 (−0.606 to −0.245)	0.064	***	<0.001
		T1 to T3	−0.558 (−0.751 to −0.366)	0.068	***	<0.001
		T1 to T4	−0.897 (−1.111 to −0.682)	0.076	***	<0.001
		T1 to T5	−0.906 (−1.130 to −0.683)	0.079	***	<0.001
		T2 to T3	−0.133 (−0.287 to 0.021)	0.055		0.16
		T2 to T4	−0.471 (−0.647 to −0.295)	0.063	***	<0.001
		T2 to T5	−0.481 (−0.671 to −0.291)	0.067	***	<0.001
		T3 to T4	−0.338 (−0.480 to −0.197)	0.050	***	<0.001
		T3 to T5	−0.348 (−0.511 to −0.185)	0.058	***	<0.001
		T4 to T5	−0.010 (−0.116 to 0.097)	0.038		>0.99
	Time × School Location (Urban)	T1 to T2	−0.350 (-0.510 to −0.190)	0.057	***	<0.001
		T1 to T3	-0.514 (−685 to −0.343)	0.061	***	<0.001
		T1 to T4	−0.810 (−1.001 to −0.620)	0.068	***	<0.001
		T1 to T5	−0.834 (−1.032 to −0.636)	0.070	***	<0.001
		T2 to T3	−0.164 (−0.301 to −0.027)	0.049	**	0.008
		T2 to T4	−0.460 (−0.617 to −0.304)	0.056	***	<0.001
		T2 to T5	−0.484 (−0.652 to −0.315)	0.060	***	<0.001
		T3 to T4	−0.296 (−0.422 to −0.170)	0.045	***	<0.001
		T3 to T5	−0.320 (−0.464 to -0.175)	0.051	***	<0.001
		T4 to T5	−0.024 (−0.118 to 0.071)	0.034		>0.99
		T1 to T2	−0.215 (−0.411 to −0.020)	0.069	*	0.020
		T1 to T3	−0.291 (−0.499 to −0.082)	0.074	**	0.001

		T1 to T4	−0.356 (−0.588 to −0.123)	0.083	***	<0.001
		T1 to T5	−0.438 (−0.681 to −0.196)	0.086	***	<0.001
		T2 to T3	−0.075 (−0.243 to 0.092)	0.059		>0.99
	Time × School Location (Rural)	T2 to T4	−0.140 (-0.331 to 0.051)	0.068		0.39
		T2 to T5	−0.223 (−0.429 to −0.017)	0.073	*	0.023
		T3 to T4	−0.065 (−0.219 to 0.089)	0.055		>0.99
		T3 to T5	−0.148 (−0.324 to 0.029)	0.063		0.19
		T4 to T5	−0.083 (−0.198 to 0.033)	0.041		0.44

a = adjusted for multiple comparisons using Bonferroni correction. p-lvl (*p*-value level) * = $p < 0.05$, ** = $p < 0.01$, *** = $p < 0.001$, BMI = body mass index, CI = confidence interval, EQUI BMI_{AUT} = equivalent BMI based on Austrian reference centile curves passing through adult BMI values [1], Mean diff = mean difference based on the estimated marginal means, p-lvl = significance level, SDS = standard deviation score, SE = standard error, T1 = baseline measurements in September 2019, T2 = follow-up measurements in June 2020, T3 = follow-up measurements in September 2020, T4 = follow-up measurements in Mar 2021, T5 = follow-up measurements in June 2021.

Table S7. Post hoc tests for waist-to-height ratio for the main effect time and interactions for time × sex and time × school location based on the estimated marginal means.

Variable	Subgroups	Pairwise comparisons	Mean diff (95% CI)	SE	p-lvl	*p*-Value[a]
WHtR	Time	T1 to T2	−0.016 (−0.020 to −0.013)	0.001	***	<0.001
		T1 to T3	0.012 (0.009 to 0.016)	0.001	***	<0.001
		T1 to T4	−0.009 (−0.013 to −0.005)	0.001	***	<0.001
		T1 to T5	0.029 (0.025 to 0.033)	0.001	***	<0.001
		T2 to T3	0.029 (0.025 to 0.033)	0.001	***	<0.001
		T2 to T4	0.007 (0.004 to 0.011)	0.001	***	<0.001
		T2 to T5	0.045 (0.042 to 0.049)	0.001	***	<0.001
		T3 to T4	−0.022 (−0.025 to −0.018)	0.001	***	<0.001
		T3 to T5	0.016 (0.013 to 0.020)	0.001	***	<0.001
		T4 to T5	0.038 (0.035 to 0.041)	0.001	***	<0.001
	Time × Sex (Girls)	T1 to T2	−0.015 (−0.021 to −0.010)	0.002	***	<0.001
		T1 to T3	0.016 (0.011 to 0.021)	0.002	***	<0.001
		T1 to T4	−0.004 (−0.010 to 0.001)	0.002		0.27
		T1 to T5	0.036 (0.031 to 0.041)	0.002	***	<0.001
		T2 to T3	0.031 (0.026 to 0.036)	0.002	***	<0.001
		T2 to T4	0.011 (0.006 to 0.016)	0.002	***	<0.001
		T2 to T5	0.051 (0.046 to 0.056)	0.002	***	<0.001
		T3 to T4	−0.020 (−0.025 to −0.015)	0.002	***	<0.001
		T3 to T5	0.020 (0.015 to 0.025)	0.002	***	<0.001
		T4 to T5	0.040 (0.036 to 0.045)	0.002	***	<0.001
	Time × Sex (Boys)	T1 to T2	−0.018 (−0.024 to −0.012)	0.002	***	<0.001
		T1 to T3	0.009 (0.004 to 0.014)	0.002	***	<0.001
		T1 to T4	−0.014 (−0.020 to −0.009)	0.002	***	<0.001
		T1 to T5	0.022 (0.016 to 0.027)	0.002	***	<0.001
		T2 to T3	0.027 (0.022 to 0.032)	0.002	***	<0.001
		T2 to T4	0.004 (−0.001 to 0.009)	0.002		0.32
		T2 to T5	0.039 (0.034 to 0.045)	0.002	***	<0.001
		T3 to T4	−0.023 (−0.028 to −0.018)	0.002	***	<0.001
		T3 to T5	0.012 (0.007 to 0.017)	0.002	***	<0.001
		T4 to T5	0.036 (0.031 to 0.040)	0.002	***	<0.001
		T1 to T2	−0.027 (−0.032 to −0.022)	0.002	***	<0.001
		T1 to T3	0.002 (−0.002 to 0.007)	0.002		>0.99

	Time × School Location (Urban)	T1 to T4	−0.020 (−0.025 to −0.016)	0.002	***	<0.001
		T1 to T5	0.018 (0.013 to 0.023)	0.002	***	<0.001
		T2 to T3	0.029 (0.025 to 0.034)	0.002	***	<0.001
		T2 to T4	0.006 (0.002 to 0.011)	0.002	**	0.001
		T2 to T5	0.045 (0.040 to 0.050)	0.002	***	<0.001
		T3 to T4	−0.023 (−0.027 to −0.018)	0.002	***	<0.001
		T3 to T5	0.016 (0.011 to 0.020)	0.002	***	<0.001
		T4 to T5	0.039 (0.035 to 0.043)	0.001	***	<0.001
	Time × School Location (Rural)	T1 to T2	−0.006 (−0.012 to 0.000)	0.002	*	0.037
		T1 to T3	0.022 (0.017 to 0.028)	0.002	***	<0.001
		T1 to T4	0.002 (−0.004 to 0.008)	0.002		>0.99
		T1 to T5	0.039 (0.033 to 0.045)	0.002	***	<0.001
		T2 to T3	0.029 (0.023 to 0.034)	0.002	***	<0.001
		T2 to T4	0.008 (0.003 to 0.014)	0.002	***	<0.001
		T2 to T5	0.046 (0.040 to 0.051)	0.002	***	<0.001
		T3 to T4	−0.020 (−0.026 to −0.015)	0.002	***	<0.001
		T3 to T5	0.017 (0.012 to 0.022)	0.002	***	<0.001
		T4 to T5	0.037 (0.032 to 0.042)	0.002	***	<0.001

a = adjusted for multiple comparisons using Bonferroni correction. p-lvl (*p*-value level) * = $p < 0.05$, ** = $p < 0.01$, *** = $p < 0.001$, BMI = body mass index, CI = confidence interval, WHtR = waist-to-height ratio, Mean diff = mean difference based on the estimated marginal means, p-lvl = significance level, SDS = standard deviation score, SE = standard error, T1 = baseline measurements in September 2019, T2 = follow-up measurements in June 2020, T3 = follow-up measurements in September 2020, T4 = follow-up measurements in Mar 2021, T5 = follow-up measurements in June 2021.

Table S8. Post hoc tests for fitness tests for the main effect time and interactions for time × sex, time × school location and time*school grade based on the estimated marginal means.

Variable	Subgroups	Pairwise comparisons	Mean diff (95% CI)	SE	p-lvl	*p*-value[a]
**6MR SDS (DüMo)**	Time	T1 to T3	1.084 (1.000 to 1.168)	0.035	***	<0.001
		T1 to T5	0.921 (0.832 to 1.010)	0.037	***	<0.001
		T3 to T5	−0.163 (−0.244 to −0.083)	0.034	***	<0.001
	Time × Sex (Girls)	T1 to T3	1.063 (0.945 to 1.180)	0.049	***	<0.001
		T1 to T5	0.988 (0.863 to 1.112)	0.052	***	<0.001
		T3 to T5	−0.075 (−0.188 to 0.038)	0.047		0.34
	Time × Sex (Boys)	T1 to T3	1.106 (0.986 to 1.226)	0.050	***	<0.001
		T1 to T5	0.854 (0.727 to 0.981)	0.053	***	<0.001
		T3 to T5	−0.252 (−0.367 to −0.137)	0.048	***	<0.001
**SLJ SDS (DüMo)**	Time	T1 to T3	−0.209 (−0.286 to −0.131)	0.032	***	<0.001
		T1 to T5	−0.079 (−0.157 to −0.001)	0.033	*	0.047
		T3 to T5	0.130 (0.059 to 0.202)	0.030	***	<0.001
	Time × School Location (Urban)	T1 to T3	−0.052 (−0.150 to 0.046)	0.041		0.62
		T1 to T5	0.003 (−0.096 to 0.102)	0.041		>0.99
		T3 to T5	0.055 (−0.036 to 0.146)	0.038		0.44
	Time × School Location (Rural)	T1 to T3	−0.366 (−0.486 to −0.246)	0.050	***	<0.001
		T1 to T5	−0.161 (−0.282 to −0.040)	0.050		0.004
		T3 to T5	0.205 (0.094 to 0.316)	0.046	***	<0.001
	Time × School Class (SY 19-20—grade 2)	T1 to T3	−0.409 (−0.520 to −0.297)	0.046	***	<0.001
		T1 to T5	−0.328 (−0.440 to −0.216)	0.047	***	<0.001
		T3 to T5	0.081 (-0.022 to 0.183)	0.043		0.18
		T1 to T3	−0.009 (−0.117 to 0.099)	0.045		>0.99

	Time × School Class (SY 19-20—grade 3)	T1 to T5	0.170 (0.062 to 0.279)	0.045	**	0.001
		T3 to T5	0.179 (0.080 to 0.279)	0.041	***	<0.001
**MB1kg (KATS-K)**	Time	T1 to T3	0.095 (0.016 to 0.174)	0.033	*	0.012
		T1 to T5	−0.023 (−0.110 to 0.063)	0.036		>0.99
		T3 to T5	−0.118 (-0.194 to −0.042)	0.032	**	0.001
	Time × Sex (Girls)	T1 to T3	-0.045 (−0.154 to 0.065)	0.045		0.98
		T1 to T5	-0.099 (−0.220 to 0.021)	0.050		0.15
		T3 to T5	-0.055 (−0.160 to 0.051)	0.044		0.64
	Time × Sex (Boys)	T1 to T3	0.234 (0.121 to 0.348)	0.047	***	<0.001
		T1 to T5	0.053 (−0.073 to 0.178)	0.052		0.94
		T3 to T5	−0.182 (−0.291 to −0.073)	0.046	***	<0.001
**4 x 10 SHR (MCA)**	Time	T1 to T3	0.307 (0.225 to 0.389)	0.034	***	<0.001
		T1 to T5	−0.357 (−0.436 to −0.277)	0.033	***	<0.001
		T3 to T5	−0.663 (−0.742 to −0.585)	0.033	***	<0.001
	Time × School Location (Urban)	T1 to T3	0.503 (0.399 to 0.608)	0.043	***	<0.001
		T1 to T5	-0.182 (-0.283 to −0.081)	0.042	***	<0.001
		T3 to T5	−0.685 (−0.784 to −0.586)	0.041	***	<0.001
	Time × School Location (Rural)	T1 to T3	0.111 (-0.017 to −0.238)	0.053		0.11
		T1 to T5	−0.531 (-0.654 to −0.408)	0.051	***	<0.001
		T3 to T5	−0.641 (-0.762 to −0.521)	0.050	***	<0.001

a = adjusted for multiple comparisons using Bonferroni correction. Mean diff = mean difference based on the estimated marginal means, CI = confidence interval, SE = standard error, p-lvl (*p*-value level) * = $p < 0.05$, ** = $p < 0.01$, *** = $p < 0.001$, p-lvl = significance level, 6MR = 6 minute run, SLJ = standing long jump, MB1kg = medicine ball throw (1 kilogram), 4 x 10 SHR = 4 x 10 meter shuttle run, SDS = standard deviation score, z-value = traditional z-score standardization; DüMo = Düsseldorfer Modell [2], KATS-K = Karlsruher test system [3], MCA = Motor Competence Assessment [4], SY 19-20—grade 2 = children visiting in school year 2019/20 grade 2 of primary school, SY 19-20—grade 3 = children visiting in school year 2019/20 grade 3 of primary school, T1 = baseline measurements in September 2019, T3 = follow-up measurements in September 2020, T5 = follow-up measurements in June 2021.

Table S9. Friedman test for weight classification and estimation of health risk using cut-offs described in AUT FIT to baseline measurements T1 and follow-up measurements T2, T3, T4, and T5.

Variable	Subgroup	df	X^2	p-lvl	*p*-Value
**AUT weight classification**	All (*n* = 708)	4	102.305	***	<0.001
	Girls (*n* = 350)	4	19.760	**	0.001
	Boys (*n* = 358)	4	94.573	***	<0.001
	Urban schools (*n* = 424)	4	97.118	*	0.012
	Rural school (*n* = 284)	4	12.857	***	<0.001
**WHtR estimation of health risk**	All (*n* = 708)	4	194.750	***	<0.001
	Girls (*n* = 350)	4	103.406	***	<0.001
	Boys (*n* = 358)	4	108.752	***	<0.001
	Urban schools (*n* = 424)	4	142.724	***	<0.001
	Rural school (*n* = 284)	4	72.701	***	<0.001

n = Study population, df = degrees of freedom, X2 = chi-square test value, p-lvl (*p*-value level) * = $p < 0.05$, ** = $p < 0.01$, *** = $p < 0.001$, p-lvl = significance level, AUT weight classification = based on Austrian reference centile curves passing through adult BMI values [1], WHtR = waist-to-height ratio, T1 = baseline measurements in September 2019, T2 = follow-up measurements in June 2020, T3 = follow-up measurements in September 2020, T4 = follow-up measurements in Mar 2021, T5 = follow-up measurements in June 2021.

Table S10. Post hoc analyses by the Wilcoxon test for weight classifications using Austrian cut-offs.

Variable	Subgroup	Pairwise comparisons	Z[a,b]	p-lvl	*p*-Value[c]
**AUT weight classification**	All	T1 to T2	−3.488[a]	**	0.005
		T1 to T3	−6.257[a]	***	<0.001
		T1 to T4	−6.734[a]	***	<0.001
		T1 to T5	−7.436[a]	***	<0.001
		T2 to T3	−3.153[a]	**	0.016
		T2 to T4	−4.388[a]	***	<0.001
		T2 to T5	−5.039[a]	***	<0.001
		T3 to T4	−1.692[a]		0.91
		T3 to T5	−2.605[a]		0.09
		T4 to T5	−1.234[a]		>0.99
	Girls	T1 to T2	−1.976[a]		0.48
		T1 to T3	−3.530[a]	**	0.004
		T1 to T4	−2.722[a]		0.06
		T1 to T5	−3.618[a]	**	0.003
		T2 to T3	−1.219[a]		>0.99
		T2 to T4	−1.061[a]		>0.99
		T2 to T5	−2.030[a]		0.42
		T3 to T4	−0.180[b]		>0.99
		T3 to T5	−1.029[a]		>0.99
		T4 to T5	−1.460[a]		>0.99
	Boys	T1 to T2	−2.887[a]	*	0.039
		T1 to T3	−5.166[a]	***	<0.001
		T1 to T4	−6.374[a]	***	<0.001
		T1 to T5	−6.644[a]	***	<0.001
		T2 to T3	−3.212[a]	*	0.013
		T2 to T4	−4.814[a]	***	<0.001
		T2 to T5	−5.013[a]	***	<0.001
		T3 to T4	−2.646[a]		0.08
		T3 to T5	−2.694[a]		0.07
		T4 to T5	−0.229[a]		>0.99
	Urban	T1 to T2	−3.414[a]	**	0.006
		T1 to T3	−5.750[a]	***	<0.001
		T1 to T4	−6.336[a]	***	<0.001
		T1 to T5	−7.272[a]	***	<0.001
		T2 to T3	−3.086[a]	*	0.020
		T2 to T4	−4.243[a]	***	<0.001
		T2 to T5	−5.082[a]	***	<0.001
		T3 to T4	−1.622[a]		>0.99
		T3 to T5	−2.655[a]		0.08
		T4 to T5	−1.300[a]		>0.99
	Rural	T1 to T2	−1.091[a]		>0.99
		T1 to T3	−2.524[a]		0.12
		T1 to T4	−2.556[a]		0.11
		T1 to T5	−2.535[a]		0.11
		T2 to T3	−1.177[a]		>0.99
		T2 to T4	−1.671[a]		0.95
		T2 to T5	−1.715[a]		0.86

		T3 to T4	−0.655[a]		>0.99
		T3 to T5	−0.816[a]		>0.99
		T4 to T5	−0.277[a]		>0.99

a = Based on positive ranks, b = based on negative ranks, c = adjusted for multiple comparisons using Bonferroni correction, c = based on positive ranks. Study population, n = 708; Z = test statistic; p-lvl (*p*-value level) * = $p < 0.05$, ** = $p < 0.01$, *** = $p < 0.001$, p-lvl = significance level, AUT weight classification = based on Austrian reference centile curves passing through adult BMI values [1], T1 = baseline measurements in September 2019, T2 = follow-up measurements in June 2020, T3 = follow-up measurements in September 2020, T4 = follow-up measurements in Mar 2021, T5 = follow-up measurements in June 2021.

Table S11. Post hoc analyses by the Wilcoxon test for estimation of health risk using cut-offs described in AUT FIT.

Variable	Subgroup	Pairwise comparisons	Z[a,b]	p-lvl	*p*-Value[c]
WHtR, estimation of health risk	All	T1 to T2	−6.749[a]	***	<0.001
		T1 to T3	−0.834[b]		>0.99
		T1 to T4	−6.139[a]	***	<0.001
		T1 to T5	−4.412[b]	***	<0.001
		T2 to T3	−7.720[b]	***	<0.001
		T2 to T4	−0.847[b]		>0.99
		T2 to T5	−10.310[b]	***	<0.001
		T3 to T4	−7.506[a]	***	<0.001
		T3 to T5	−4.041[b]	***	<0.001
		T4 to T5	−9.798[b]	***	<0.001
	Girls	T1 to T2	−3.395[a]	**	0.007
		T1 to T3	−3.000[b]	*	0.027
		T1 to T4	−2.292[a]		0.22
		T1 to T5	−5.431[b]	***	<0.001
		T2 to T3	−5.986[b]	***	<0.001
		T2 to T4	−1.457[b]		>0.99
		T2 to T5	−7.665[b]	***	<0.001
		T3 to T4	−5.171[a]	***	<0.001
		T3 to T5	−3.086[b]	*	0.020
		T4 to T5	−6.742[b]	***	<0.001
	Boys	T1 to T2	−6.140[a]	***	<0.001
		T1 to T3	−1.982[a]		0.47
		T1 to T4	−6.120[a]	***	<0.001
		T1 to T5	−0.324[b]		>0.99
		T2 to T3	−4.906[b]	***	<0.001
		T2 to T4	−0.267[a]		>0.99
		T2 to T5	−6.900[b]	***	<0.001
		T3 to T4	−5.443[a]	***	<0.001
		T3 to T5	−2.611[b]		0.09
		T4 to T5	−7.160[b]	***	<0.001
	Urban	T1 to T2	−7.507[a]	***	<0.001
		T1 to T3	−1.540[a]		>0.99
		T1 to T4	−6.724[a]	***	<0.001
		T1 to T5	−0.866[b]		>0.99
		T2 to T3	−6.496[b]	***	<0.001
		T2 to T4	−1.116[b]		>0.99
		T2 to T5	−8.273[b]	***	<0.001

	T3 to T4	−5.580[a]	***	<0.001
	T3 to T5	−2.480[b]		0.13
	T4 to T5	−7.506[b]	***	<0.001
Rural	T1 to T2	−1.483[a]		>0.99
	T1 to T3	−2.967[b]	*	0.030
	T1 to T4	−1.511[a]		>0.99
	T1 to T5	−5.397[b]	***	<0.001
	T2 to T3	−4.243[b]	***	<0.001
	T2 to T4	0.000		>0.99
	T2 to T5	−6.194[b]	***	<0.001
	T3 to T4	−5.145[a]	***	<0.001
	T3 to T5	−3.402[b]	**	0.007
	T4 to T5	−6.303[b]	***	<0.001

a = Based on positive ranks, b = based on negative ranks, c = adjusted for multiple comparisons using Bonferroni correction, c = based on positive ranks. Study population, n = 708; Z = test statistic; p-lvl (*p*-value level) * = $p < 0.05$, ** = $p < 0.01$, *** = $p < 0.001$, p-lvl = significance level, WHtR = waist-to-height ratio, T1 = baseline measurements in September 2019, T2 = follow-up measurements in June 2020, T3 = follow-up measurements in September 2020, T4 = follow-up measurements in Mar 2021, T5 = follow-up measurements in June 2021.

Table S12. Overview of fitness test STA9 classifications in relation to the results of international reference values.

Fitness performance	STA9 Classification, 6MR (DüMo), No. (%) (*n* = 706)			STA9 Classification, SLJ (DüMo), No. (%) (*n* = 706)			STA9 Classification, MB1kg (KATS-K), No. (%) (*n* = 662)			STA9 Classification, 4 x 10 SHR (MCA), No. (%) (*n* = 706)		
	T1	T3	T5	T1	T3	T5	T1	T3	T5	T1	T3	T5
**Poor**	28 (4.0%)	116 (16.4%)	120 (17.0%)	44 (6.2%)	33 (4.7%)	46 (6.5%)	33 (5.0%)	31 (4.7%)	25 (3.8%)	76 (10.8%)	138 (19.5%)	61 (8.6%)
**Very weak**	34 (4.8%)	123 (17.4%)	95 (13.5%)	40 (5.7%)	34 (4.8%)	45 (6.4%)	49 (7.4%)	64 (9.7%)	50 (7.6%)	103 (14.6%)	136 (19.3%)	60 (8.5%)
**Weak**	81 (11.5%)	151 (21.4%)	118 (16.7%)	99 (14.0%)	68 (9.6%)	81 (11.5%)	94 (14.2%)	97 (14.7%)	94 (14.2%)	118 (16.7%)	144 (20.4%)	93 (13.2%)
**Below average**	99 (14.0%)	124 (17.6%)	127 (18.0%)	141 (20.0%)	130 (18.4%)	133 (18.8%)	137 (20.7%)	141 (21.3%)	133 (20.1%)	157 (22.2%)	119 (16.9%)	133 (18.8%)
**Average**	104 (14.7%)	95 (13.5%)	101 (14.3%)	110 (15.6%)	130 (18.8%)	118 (16.7%)	132 (19.9%)	144 (21.8%)	145 (21.9%)	135 (19.1%)	88 (12.5%)	149 (21%)
**Above average**	116 (16.4%)	62 (8.8%)	77 (10.9%)	113 (16.0%)	130 (18.8%)	114 (16.1%)	105 (15.9%)	96 (14.5%)	109 (16.5%)	72 (10.2%)	52 (7.4%)	110 (15.6%)

**Very well**	109 (15.4%)	25 (3.5%)	38 (5.4%)	80 (11.3%)	82 (11.6%)	86 (12.2%)	61 (9.2%)	47 (7.1%)	43 (6.5%)	34 (4.8%)	21 (3.0%)	66 (9.3%)
**Excellent**	70 (9.9%)	9 (1.3)	21 (3.0%)	53 (7.5%)	53 (7.5%)	50 (7.1%)	36 (5.4%)	22 (3.3%)	41 (6.2%)	10 (1.4%)	6 (0.8%)	24 (3.4%)
**Outstanding**	65 (9.2%)	1 (0.1%)	9 (1.3%)	26 (3.7%)	40 (5.7%)	33 (4.7%)	15 (2.3%)	20 (3.0%)	22 (3.3%)	1 (0.1%)	2 (0.3%)	10 (1.4%)

Data are No (%), 6MR DüMo = Ra. of 6 minute run by u.STA9 based on reference values from the Düsseldorfer Modell [2], SLJ DüMo = Ra. of standing long jump by u.STA9 based on reference values from the Düsseldorfer Modell [2], MB1kg KATS-K = Ra. of medicine ball throw 1 kg by u.STA9 based on reference values from the Karlsruher test system [3], 4-m × 10-m SHR MCA = Ra. of 4-m × 10-m shuttle run by u.STA9 based on Portuguese reference values [4], m = meter, u.STA9 = using a nine-point scale, Ra. = Ranking, *n* = number of participations, T1 = baseline measurements in September 2019, T3 = follow-up measurements in September 2020, T5 = follow-up measurements in June 2021.

Table S13. Friedman test for fitness performance categories using cut-offs described in AUT FIT to baseline measurements T1 and follow-up measurements T3 and T5.

Variable	Subgroup	df	X^2	p-lvl	*p*-Value
**STA9 Classification, 6MR (DüMo)**	All (*n* = 706)	2	598.451	***	<0.001
	Girls (*n* = 349)	2	299.961	***	<0.001
	Boys (*n* = 357)	2	302.576	***	<0.001
	Urban schools (*n* = 423)	2	347.021	***	<0.001
	Rural school (*n* = 283)	2	253.492	***	<0.001
	SY 19-20—grade 2 (*n* = 345)	2	239.289	***	<0.001
	SY 19-20—grade 3 (*n* = 361)	2	367.385	***	<0.001
**STA9 Classification, SLJ (DüMo)**	All (*n* = 706)	2	36.183	***	<0.001
	Girls (*n* = 349)	2	25.458	***	<0.001
	Boys (*n* = 357)	2	12.624	**	0.002
	Urban schools (*n* = 423)	2	5.245		0.07
	Rural school (*n* = 283)	2	48.852	***	<0.001
	SY 19-20—grade 2 (*n* = 345)	2	63.186	***	<0.001
	SY 19-20—grade 3 (*n* = 361)	2	30.481	***	<0.001
**STA9 Classification, MB1kg (KATS-K)**	All (*n* = 662)	2	12.063	**	0.002
	Girls (*n* = 330)	2	3.067		0.22
	Boys (*n* = 332)	2	21.230	***	<0.001
	Urban schools (*n* = 394)	2	6.936		0.03
	Rural school (*n* = 268)	2	7.136	*	0.028
	SY 19-20—grade 2 (*n* = 344)	2	0.185		0.91
	SY 19-20—grade 3 (*n* = 318)	2	25.502	***	<0.001
**STA9 Classification, 4-m × 10-m SHR SDS (MCA)**	All (*n* = 706)	2	285.022	***	<0.001
	Girls (*n* = 349)	2	167.663	***	<0.001
	Boys (*n* = 357)	2	121.553	***	<0.001
	Urban schools (*n* = 423)	2	194.778	***	<0.001
	Rural school (*n* = 283)	2	110.433	***	<0.001
	SY 19-20—grade 2 (*n* = 345)	2	104.484	***	<0.001
	SY 19-20—grade 3 (*n* = 361)	2	190.197	***	<0.001

n = Study population, df = degrees of freedom, X^2 = chi-square test value, p-lvl (*p*-value level) * = $p < 0.05$, ** = $p < 0.01$, *** = $p < 0.001$, p-lvl = significance level, 6MR DüMo = Ra. of 6 minute run by u.STA9 based on reference values from the Düsseldorfer Modell [2], SLJ DüMo = Ra. of standing long jump by u.STA9 based on reference values from the Düsseldorfer Modell [2], MB1kg KATS-K = Ra. of medicine ball throw 1 kg by u.STA9 based on reference values from the Karlsruher test system [3], 4-m × 10-m SHR MCA = Ra. of 4-m × 10-m shuttle run by u.STA9 based on Portuguese reference values

[4], u.STA9 = using a nine-point scale, Ra. = Ranking, *n* = number of participations, SY 19-20—grade 2 = children visiting in school year 2019/20 grade 2 of primary school, SY 19-20—grade 3 = children visiting in school year 2019/20 grade 3 of primary school, m = meter, T1 = baseline measurements in September 2019, T3 = follow-up measurements in September 2020, T5 = follow-up measurements in June 2021.

Table S14. Post hoc analyses by the Wilcoxon test for fitness tests using cut-offs described in AUT FIT.

Variable	Subgroup	Pairwise comparisons	Z[a,b]	p-lvl	*p*-Value[c]
**STA9 Classification, 6MR (DüMo)**	All	T1 to T3	−20.230[a]	***	<0.001
		T1 to T5	−17.810[a]	***	<0.001
		T3 to T5	−5.302[b]	***	<0.001
**STA9 Classification, SLJ (DüMo)**	All	T1 to T3	−4.765[a]	***	<0.001
		T1 to T5	−1.197[a]		0.69
		T3 to T5	−4.397[b]	***	<0.001
	Urban	T1 to T3	−0.728[a]		>0.99
		T1 to T5	−0.585[b]		>0.99
		T3 to T5	−1.756[b]		0.24
	Rural	T1 to T3	−6.406[a]	***	<0.001
		T1 to T5	−2.520[a]	*	0.035
		T3 to T5	−4.628[b]	***	<0.001
	SY 19-20—grade 2	T1 to T3	−7.113[a]	***	<0.001
		T1 to T5	−5.877[a]	***	<0.001
		T3 to T5	−1.770[b]		0.23
	SY 19-20—grade 3	T1 to T3	−0.755[a]		>0.99
		T1 to T5	−4.533[a]	***	<0.001
		T3 to T5	−4.491[a]	***	<0.001
**STA9 Classification, MB1kg (KATS-K)**	All	T1 to T3	−2.617[a]	*	0.027
		T1 to T5	−0.935[b]		>0.99
		T3 to T5	−3.725[b]	***	<0.001
	Girls	T1 to T3	−0.790[a]		>0.99
		T1 to T5	−2.163[a]		0.09
		T3 to T5	−1.711[a]		0.26
	Boys	T1 to T3	−4.507[a]	***	<0.001
		T1 to T5	−0.962[a]		>0.99
		T3 to T5	−3.582[b]	**	0.001
**STA9 Classification, 4-m × 10-m SHR (MCA)**	All	T1 to T3	−8.720[a]	***	<0.001
		T1 to T5	−9.111[b]	***	<0.001
		T3 to T5	−15.914[b]	***	<0.001
	Urban	T1 to T3	−9.775[a]	***	<0.001
		T1 to T5	−4.412[b]	***	<0.001
		T3 to T5	−12.489[b]	***	<0.001
	Rural	T1 to T3	−1.637[a]		0.31
		T1 to T5	−8.642[b]	***	<0.001
		T3 to T5	−9.878[b]	***	<0.001

a = Based on positive ranks, b = based on negative ranks, c = adjusted for multiple comparisons using Bonferroni correction, c = based on positive ranks. Study population, n = 708; Z = test statistic; p-lvl (*p*-value level) * = *p* < 0.05, ** = *p* < 0.01, *** = *p* < 0.001, p-lvl = significance level, 6MR DüMo = Ra. of 6 minute run by u.STA9 based on reference values from the Düsseldorfer Modell [2], SLJ DüMo = Ra. of standing long jump by u.STA9 based on reference values from the Düsseldorfer Modell [2], MB1kg KATS-K = Ra. of medicine ball throw 1 kg by u.STA9 based on reference values from the Karlsruher test system [3], 4-m × 10-m SHR MCA = Ra. of 4-m × 10-m shuttle run by u.STA9 based on Portuguese

reference values [4], u.STA9 = using a nine-point scale, Ra. = Ranking, n = number of participations, SY 19-20—grade 2 = children visiting in school year 2019/20 grade 2 of primary school, SY 19-20—grade 3 = children visiting in school year 2019/20 grade 3 of primary school, m = meter, T1 = baseline measurements in September 2019, T3 = follow-up measurements in September 2020, T5 = follow-up measurements in June 2021.

Figure S1. COVID-19 restrictions in Austria between January 31, 2020 and June 30, 2021.

A. Restriction levels for primary school children.

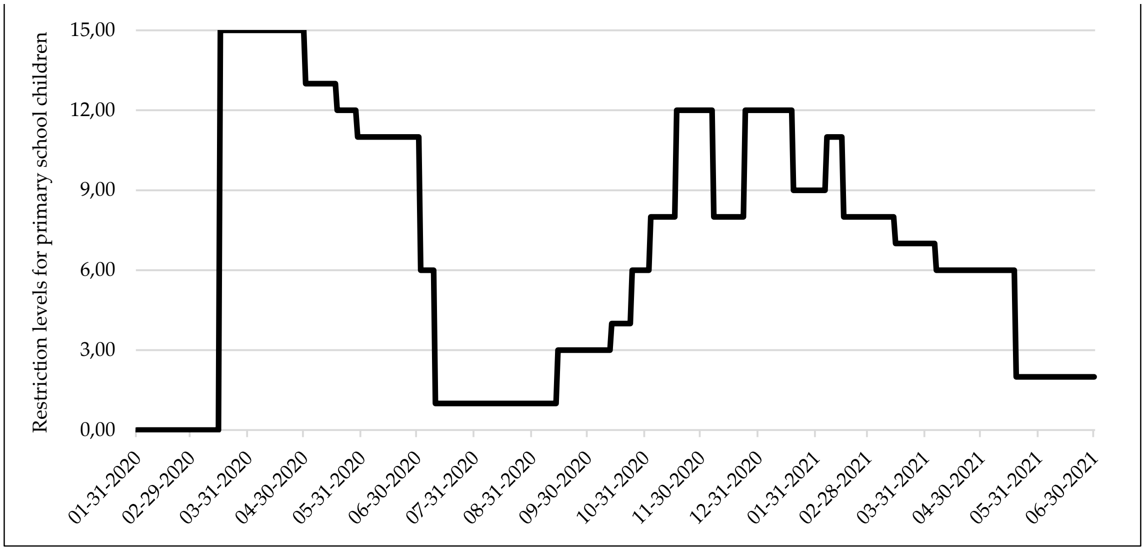

B. OxCGRT Stringency Index.

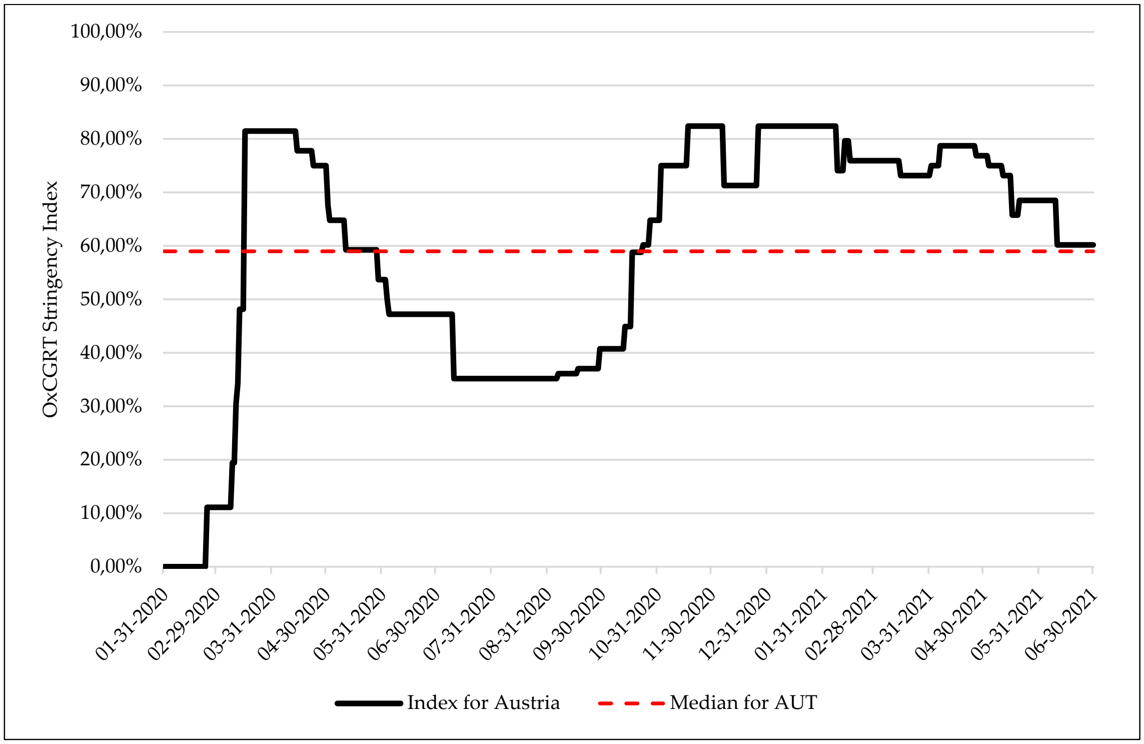

The detailed description of this classification method used in Figure S1A is available in Table S1 and Table S2. Figure S1B shows the Oxford COVID-19 Government Response Tracker (OxCGRT) Stringency Index for Austria between January 31, 2020 and June 30, 2021.

References

1. Mayer, M.; Gleiss, A.; Häusler, G.; Borkenstein, M.; Kapelari, K.; Köstl, G.; Lassi, M.; Schemper, M.; Schmitt, K.; Blümel, P. Weight and body mass index (BMI): current data for Austrian boys and girls aged 4 to under 19 years. *Ann. Hum. Biol.* **2015**, *42*, 45–55, doi:10.3109/03014460.2014.907444.
2. Stemper, T.; Bachmann, C.; Diehlmann, K.; Kemper, B. *DüMo Düsseldorfer Modell der Bewegungs-, Sport- und Talentförderung*: *2003 - 2018: Konzept, Normwerte, Untersuchungsergebnisse;* LIT: Berlin, 2020, ISBN 9783643147783.
3. Bös, K.; Opper, E.; Woll, A.; Liebisch, R.; Breithecker, D.; Kremer, B. Das Karlsruher Testsystem für Kinder (KATS-K) - Testmanual. *Haltung und Bewegung* **2001**, *21*, 4.
4. Rodrigues, L.P.; Luz, C.; Cordovil, R.; Bezerra, P.; Silva, B.; Camões, M.; Lima, R. Normative values of the motor competence assessment (MCA) from 3 to 23 years of age. *J. Sci. Med. Sport* **2019**, *22*, 1038–1043, doi:10.1016/j.jsams.2019.05.009.
